# Maize Field Study Reveals Covaried Microbiota and Metabolic Changes in Roots over Plant Growth

**DOI:** 10.1128/mbio.02584-21

**Published:** 2022-03-08

**Authors:** Amelia Bourceret, Rui Guan, Kristof Dorau, Tim Mansfeldt, Amin Omidbakhshfard, David B. Medeiros, Alisdair R. Fernie, Joerg Hofmann, Uwe Sonnewald, Jochen Mayer, Nina Gerlach, Marcel Bucher, Ruben Garrido-Oter, Stijn Spaepen, Paul Schulze-Lefert

**Affiliations:** a Department of Plant Microbe Interactions, Max Planck Institute for Plant Breeding Research, Cologne, Germany; b Institute of Geography, Department of Geosciences, Faculty of Mathematics and Natural Sciences, University of Cologne, Cologne, Germany; c Max Planck Institute of Molecular Plant Physiology, Potsdam-Golm, Germany; d Division of Biochemistry, Department Biology, Friedrich Alexander Universität Erlangen-Nuremberg, Erlangen, Germany; e Agroscope Reckenholz-Tänikon Research Station, Zürich, Switzerland; f Institute for Plant Sciences, Cologne Biocenter, University of Cologne, Cologne, Germany; g Cluster of Excellence on Plant Sciences, Düsseldorf, Germany; University of California, Los Angeles; University of Hawaii at Manoa

**Keywords:** root microbiota, maize, soil management, root metabolome, phosphate, plant-microbe interaction, phosphate metabolism

## Abstract

Plant roots are colonized by microorganisms from the surrounding soil that belong to different kingdoms and form a multikingdom microbial community called the root microbiota. Despite their importance for plant growth, the relationship between soil management, the root microbiota, and plant performance remains unknown. Here, we characterize the maize root-associated bacterial, fungal, and oomycetal communities during the vegetative and reproductive growth stages of four maize inbred lines and the *pht1*;*6* phosphate transporter mutant. These plants were grown in two long-term experimental fields under four contrasting soil managements, including phosphate-deficient and -sufficient conditions. We showed that the maize root-associated microbiota is influenced by soil management and changes during host growth stages. We identified stable bacterial and fungal root-associated taxa that persist throughout the host life cycle. These taxa were accompanied by dynamic members that covary with changes in root metabolites. We observed an inverse stable-to-dynamic ratio between root-associated bacterial and fungal communities. We also found a host footprint on the soil biota, characterized by a convergence between soil, rhizosphere, and root bacterial communities during reproductive maize growth. Our study reveals the spatiotemporal dynamics of the maize root-associated microbiota and suggests that the fungal assemblage is less responsive to changes in root metabolites than the bacterial community.

## INTRODUCTION

In nature, plant roots are colonized by diverse soil-dwelling microbes, which are collectively known as the root microbiota, and this microbial multikingdom community promotes plant growth and health (1–4). Numerous microbiota members assist with nutrient mobilization of macro- and micronutrients from the soil for host nutrition. For instance, in orthophosphate (P)-limiting soils, P-solubilizing rhizosphere bacteria can increase the amount of bioavailable P, and the long-distance transport of soluble P is mediated by hyphae of symbiotic arbuscular mycorrhizal fungi (AMF) or certain fungal root endophytes to the host (5–7). In iron (Fe)-limiting calcareous soil, the bacterial root microbiota serves a critical role in mobilizing insoluble ferric iron for plant Fe nutrition (8). Finally, interactions between microbes from different kingdoms are important for plant survival, as shown previously by Durán et al. (9), who described the protective function of the root-associated bacterial community against a taxonomically broad range of soil-dwelling and harmful filamentous eukaryotes in Arabidopsis thaliana. Although bacteria and fungi are considered the main microbial kingdoms of the root microbiota, roots are also colonized by the kingdom Stramenopiles (formerly Oomycota), with common oomycete phytopathogenic members (10) and some strains belonging to *Pythium* spp. that are known to promote plant growth by preventing biotic stress (11).

Plant breeding allows the selection of the best traits that favor optimal plant fitness under high-fertilization conditions, but the innate capacity of inbred lines to establish beneficial plant-microbe associations has not received as much attention. The choice of long-term soil management can indirectly affect plant growth by modifying the diversity and connectivity of root-associated microbial communities (12–14). Organic fertilization appears to contribute to the maintenance of more abundant and diverse soil microbial communities (15, 16). However, little is known about the relationship of soil-dwelling microbial communities with soil nutrient status, such as how limiting soil nutrients influence the capacity of plant roots to be colonized by microbes that boost plant growth (8, 17).

Although several studies have linked temporal changes in the root-associated microbiota to plant development in both field and controlled-environment experiments, not much is known about how maize-associated microbiota are altered between plant growth stages (18–21). As one of the most widely cultivated crops in the world, maize has been used as a model to characterize plant-microbe interactions in agricultural contexts, specifically to assess the effects of plant genotype and age, biogeography, and soil management on microbial community assembly (13, 21–23). Additionally, the architecture of the maize root system is modified over time (24). Fungal communities were shown to vary for axial and lateral root types, and aerial roots of a particular maize landrace secrete a carbohydrate-rich mucilage enriched in diazotrophic bacteria (25, 26). In the crop root system, the different types of roots function in dissimilar manners regarding nutrient and water foraging and uptake (24, 27), which can affect microbial root colonization, as shown for AMF (28). However, the spatiotemporal variability of the root microbiota of maize has not been extensively explored, and few studies have considered the diverse multikingdom microbial communities associated with maize or the roots of other species in their entirety (9, 13, 23, 29).

Rhizodeposits, including soluble root exudates, represent a major source of organic carbon for soil-dwelling bacteria surrounding roots (30). Root exudates were shown to change consistently during the early vegetative and senescence developmental stages of the annual grass Avena barbata (31). Specifically, chemical succession in A. barbata exudates interacts with microbial metabolite substrate preferences in heterotrophic bacteria isolated from soil, in which *Avena* dominates (31). The age-correlated *A. barbata* exudation and microbial substrate uptake explain part of the bacterial community assembly and dynamics in the rhizosphere of this annual plant. However, in the perennial plant Arabis alpina, a comparison of vegetative and reproductive stages of the nonflowering wild type and a naturally occurring and perpetually flowering mutant did not show any impact of flowering time on root bacterial community profiles but showed a clear effect of soil residence time (32). This shows that the genetically determined program of flowering time (transition to reproductive growth) can be uncoupled from dynamic changes in the root microbiota. In the annual plant A. thaliana, root samples from young and fruiting plants clustered together, indicating that the vegetative and reproductive growth phases do not have a major effect on the overall bacterial community composition (33). In contrast, the perennial plant Boechera stricta was found to alter its root-associated bacterial community over the growing season (34). Moreover, similar results were observed in field-grown rice, presenting a shift in the bacterial and archaeal root microbiota over plant growth from the juvenile to the adult plant stages (20). Collectively, these findings suggest plant species-specific variation in root microbiota dynamics that is not necessarily linked to the genetically programmed developmental growth stages of the host.

The composition of root metabolites, which include various compound classes such as soluble carbohydrates, amino acids, fatty acids, organic acids, and specialized metabolites (35), changes during the life cycle of flowering plants (36). In maize, several studies have shown that benzoxazinoids (BXs), specialized metabolites that display insecticidal, antimicrobial, and allelopathic activities and are predominantly secreted by roots at an early growth stage, influence the root-associated microbiota by inhibiting colonization by specific microbial taxa and plant pathogens (37, 38). The BX breakdown product 6-methoxy-benzoxazolin-2-one (MBOA), which accumulates in the soil, acts indirectly by altering the root-associated microbiota and is necessary and sufficient to promote maize tolerance to herbivore attack in the next plant generation (37). In addition, BXs regulate global maize root metabolism and influence the root microbiota via BX-dependent metabolites, especially flavonoids (39).

Here, we examined how soil-dwelling and root-associated microbial communities from four maize inbred lines (B73, PH207, DK105, and F2) respond to contrasting organic (biodynamic mixed [BIODYN]) and mineral (NK [nitrogen and potassium], NPK [nitrogen, phosphate, and potassium], and conventional solely mineral-fertilized [CONMIN]) soil managements in long-term fertilization fields at two different locations (fertilization demonstration experiment [DEMO] and dynamic, organic, and conventional management [DOK] fields, respectively). In addition, considering the major role of soil orthophosphate availability in crop growth and the positive interaction between maize and AMF, we included the *pht1*;*6* P transporter mutant in our study. We surveyed 1,104 samples (of soil, rhizosphere, and root compartments, at three different time points) by amplicon sequencing of bacterial, fungal, and oomycete marker genes to reveal the dynamics of the soil and root microbiota. Comparison of maize samples collected at the vegetative and reproductive growth stages shows that the root-associated microbiota is influenced by soil management and is dynamic over the host’s life cycle. Moreover, we found a convergence of bacterial soil, rhizosphere, and root communities at the phylum level over the growing season. By performing parallel profiling of root lipids, amino acids, soluble carbohydrates, and the root ionome, we show that root metabolites covary with root-associated microbial communities. Comparison of wild-type and *pht1*;*6* mutant plants revealed a potential plant growth stage-specific link between AMF symbiosis, root lipid status, and soil P availability. We discuss the potential interplay between the root microbiota, root metabolites, and soil management over the life cycle of field-grown maize and highlight how the dynamics of plant-microbe associations could affect plant physiology and fitness depending on soil nutrient availability.

## RESULTS

### Dynamics of the soil- and root-associated microbiota diversity.

**FIG 1:**
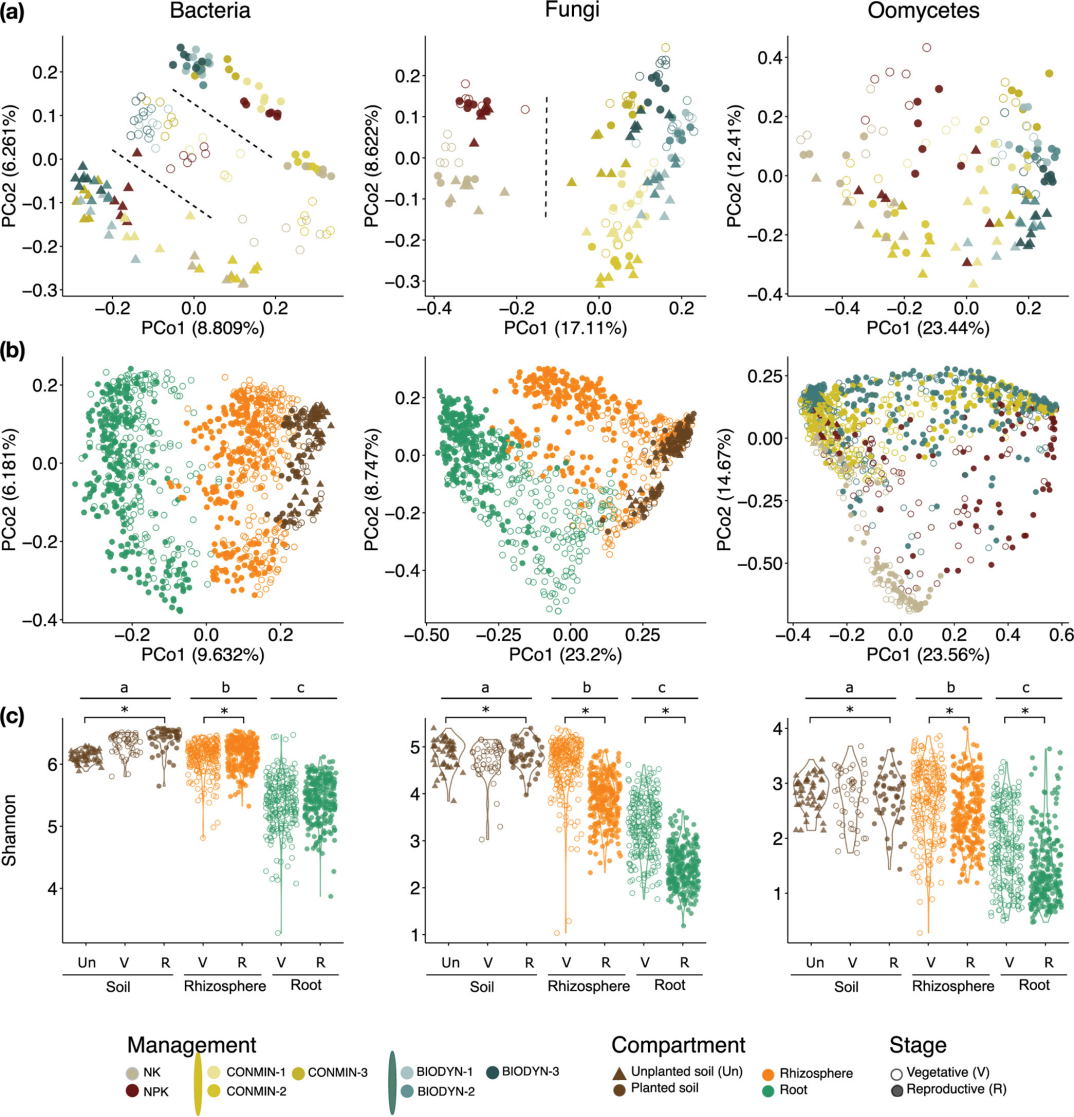
Soil physicochemical properties and management, root compartment, and plant growth phase shape the soil- and root-associated microbiota in field-grown maize. (a) PCoA based on Bray-Curtis dissimilarity between unplanted and planted soil samples at the vegetative and reproductive stages (*n* = 144). (b) PCoA of all harvested samples, including all three compartments (soil, rhizosphere, and root), four soil managements (NK; NPK; CONMIN plots 1, 2, and 3; and BIODYN plots 1, 2, and 3), and three sampling times (unplanted soil before sowing, vegetative stage, and reproductive stage) (for bacteria, fungi, and oomycetes, *n* = 1,079, 1,103, and 1,103, respectively). (c) Alpha-diversity (Shannon index) of all samples. A Wilcoxon test (*P < *0.05) was used for statistical analysis with false discovery rate (FDR) correction. Capital letters indicate significant differences between compartments, asterisks indicate significant differences between different plant growth phases within each compartment, and dashed lines indicate sample separation.

We assessed the community compositions of the three main microbial kingdoms, bacteria (B), fungi (F), and oomycetes (O), in the respective soil samples: NK and NPK in the DEMO field and CONMIN and BIODYN in the DOK field. Except for the well-known field effect on all the microbial kingdoms, an impact of soil properties on the unplanted bulk soil biota was observed (see Fig. S2 in the supplemental material), where bacterial communities of plots with the lowest pH values (5.97 ± 0.13 in NK and 5.91 ± 0.16 in CONMIN-2) (Fig. S2b) were clustered together (Fig. S2d), and microbial communities from the plots with the highest clay content (*P < *0.05) (CONMIN-3 and BIODYN-3) (Fig. S2c) exhibited lower dissimilarities in all three kingdoms (Fig. S2d to f). In planted soil, besides the field, management, and plot effects, microbial communities were also clustered by the corresponding host growth stage when sampling (Fig. 1a). Although the plant growth phase influenced the diversity of soil microbes from all three kingdoms with relatively high explained variance ratios (B, 10.10%; F, 7.28%; O, 10.71% [*P < *0.001 by permutational multivariate analysis of variance {PERMANOVA}]) (see Tables S2 to S7 in the appendix at https://github.com/Guan06/Bourceret_and_Guan_et_al_2022), this effect was more clearly observed in bacterial communities (Fig. 1a). Moreover, soil bacterial communities exhibited various alpha-diversities associated with soil chemistry and plant growth stage (Fig. S3a). The Shannon index was significantly higher in the DOK field (CONMIN and BIODYN) than in the DEMO field (NK and NPK) for bacteria (*P < *0.001).

10.1128/mBio.02584-21.3 FIG S2 Soil properties and microbial diversity of unplanted soil long-term experimental fields. (a) Principal-component analysis (PCA) of Euclidean distances of soil properties (see Table S1 in the supplemental material for details of each property) (34 properties in 48 samples were analyzed). (b and c) Variability of pH (b) and clay content (c) of the soil between plots in each field (*n* = 48) (a Wilcoxon test with FDR correction was used for statistical analysis [*P < *0.05]). (d to f) Beta-diversity (Bray-Curtis dissimilarity) of bacterial (d), fungal (e), and oomycetal (f) communities in unplanted soil collected before sowing (*n* = 48). Download FIG S2, EPS file, 0.4 MB. Copyright © 2022 Bourceret et al. 2022 Bourceret et al. https://creativecommons.org/licenses/by/4.0/ This content is distributed under the terms of the Creative Commons Attribution 4.0 International license.

10.1128/mBio.02584-21.4 FIG S3 Root compartment, soil management, and plant growth phase are the main drivers of shifts in the root-associated microbiota in field-grown maize. (a) Soil microbial alpha-diversity is impacted by plant growth phase and soil management. Shannon indices of bacterial, fungal, and oomycetal communities are shown, and the Wilcoxon test with FDR correction was used for statistical analysis. Capital letters indicate significant differences between managements; lowercase letters indicate significant differences between time points. There were three plots per management in the DOK field (*n* = 6 per plot). CO, CONMIN (conventional solely mineral); BI, BIODYN (biodynamic mixed); Un, unplanted soil before sowing; V, vegetative stage; R, reproductive stage. (b and c) PCoA of all harvested samples for the three microbial kingdoms (*n* = 1,079, 1,103, and 1,103 samples for bacteria, fungi, and oomycetes, respectively) at PCo1 and -3 (b) and PCo3 and -4 (c). (d) The explained variance (percent) of each factor was calculated by PERMANOVA. Significant factors (*P < *0.001) explaining more than 1% of the variance are shown here, and other factors are integrated into “Others.” Technical factors (TF) include batch effects of the sequencing run and all other related parameters. Download FIG S3, EPS file, 2.9 MB. Copyright © 2022 Bourceret et al. 2022 Bourceret et al. https://creativecommons.org/licenses/by/4.0/ This content is distributed under the terms of the Creative Commons Attribution 4.0 International license.

We extended the characterization of all three microbial kingdom communities to all tested compartments, including soil, rhizosphere, and root, at the vegetative and reproductive growth phases (Fig. 1b; Fig. S3b to d). Bacterial and fungal communities were clustered by compartment (Fig. 1b) and distinguished by host growth stage and management practices along the fourth and third axes, respectively (Fig. S3b and c). The oomycetal communities showed a larger dispersion and partially clustered by soil management (Fig. 1b). The main drivers observed to be responsible for variations in the soil- and root-associated microbiota were confirmed by PERMANOVA (Fig. S3d). For all microbial kingdoms, we observed a decrease in diversity (Shannon indices) from soil to rhizosphere and root (Fig. 1c). However, the bacterial alpha-diversity increased in both soil and rhizosphere from the vegetative to the reproductive growth phases while remaining stable in the root compartment over both growth stages. In contrast, for fungi and oomycetes, a decrease in diversity was observed for both rhizosphere and root over the growing season.

### Stable root-associated microbial taxa over host growth.

**FIG 2:**
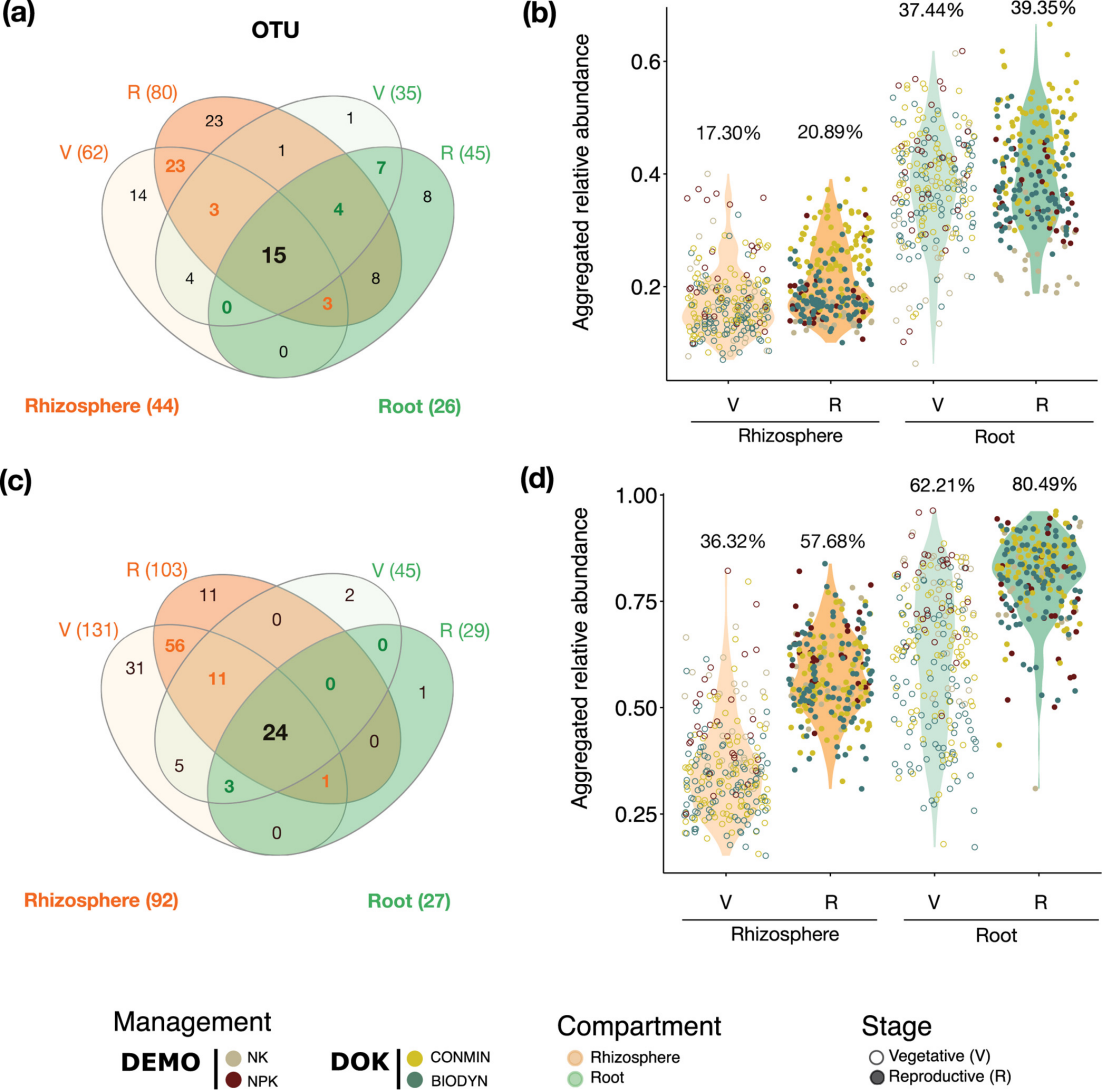
Stable bacterial and fungal OTUs are enriched from rhizosphere to root compartment over plant growth, irrespective of soil management. (a and c) Venn diagrams showing the numbers of bacterial (a) and fungal (c) OTUs found in more than 80% of samples in each compartment at both plant growth stages (including NK, NPK, CONMIN, and BIODYN soil managements and B73, DK105, PH207, and F2 plant genotypes). (b and d) Relative abundances of stable OTUs were aggregated and are demonstrated for bacteria (b) and fungi (d). The stable-to-dynamic ratio was calculated as the ratio of aRAs between stable and dynamic community members. For example, the stable aRA of root-associated bacteria at the reproductive stage is 39.35% (b); hence, the dynamic aRA is 60.65% (100% − 39.35%), and thus, the stable-to-dynamic ratio for bacteria in the root at the reproductive stage is 0.65 (60.65%/39.35%).

Despite the above-mentioned dynamics of microbial communities over the growing season, we were also able to investigate the stability of root-associated microbial members (Fig. 2; see also Appendix 3 at https://github.com/Guan06/Bourceret_and_Guan_et_al_2022) by examining the widespread (found in >80% of samples under the corresponding condition) taxa that are persistent, i.e., detected at both the vegetative and reproductive plant growth stages. We identified 26 stable bacterial operational taxonomic units (OTUs) in the root compartment (Fig. 2a), consisting of *Proteobacteria* and *Actinobacteria* (16 and 10 OTUs, respectively) and representing more than half of the entire root community (50.94% aggregated relative abundance [aRA]). Furthermore, 15 of the 26 stable OTUs were shared between the root and rhizosphere compartments. These 15 OTUs, mainly *Proteobacteria*, accounted in the rhizosphere for 17.30% and 20.89% aRAs in the vegetative and reproductive growth phases (Fig. 2b), respectively, and their aRAs increased in the root compartment (to 37.44% and 39.35%, respectively), independent of field location or soil management (Fig. 2b). This finding indicates their progressive enrichment when moving from rhizosphere to root compartments. A similar pattern was found for the fungal community. We identified 24 stable fungal OTUs between the rhizosphere and root compartments (Fig. 2c), irrespective of management practices and field location, representing on average an 80.49% aRA in reproductive roots (Fig. 2d). Most of these members were affiliated with four classes of Ascomycota (23/24 OTUs), namely, Dothideomycetes, Eurotiomycetes, Leotiomycetes, and Sordariomycetes (see Tables S2 to S7 at the URL mentioned above). Interestingly, we found an inverse stable-to-dynamic ratio of bacterial and fungal root-associated assemblages (0.65 and 4.13 in roots at the reproductive stage, respectively) (the calculation is described in the Fig. 2 legend).

### Microbial community assembly patterns at different phylogenetic levels.

**FIG 3:**
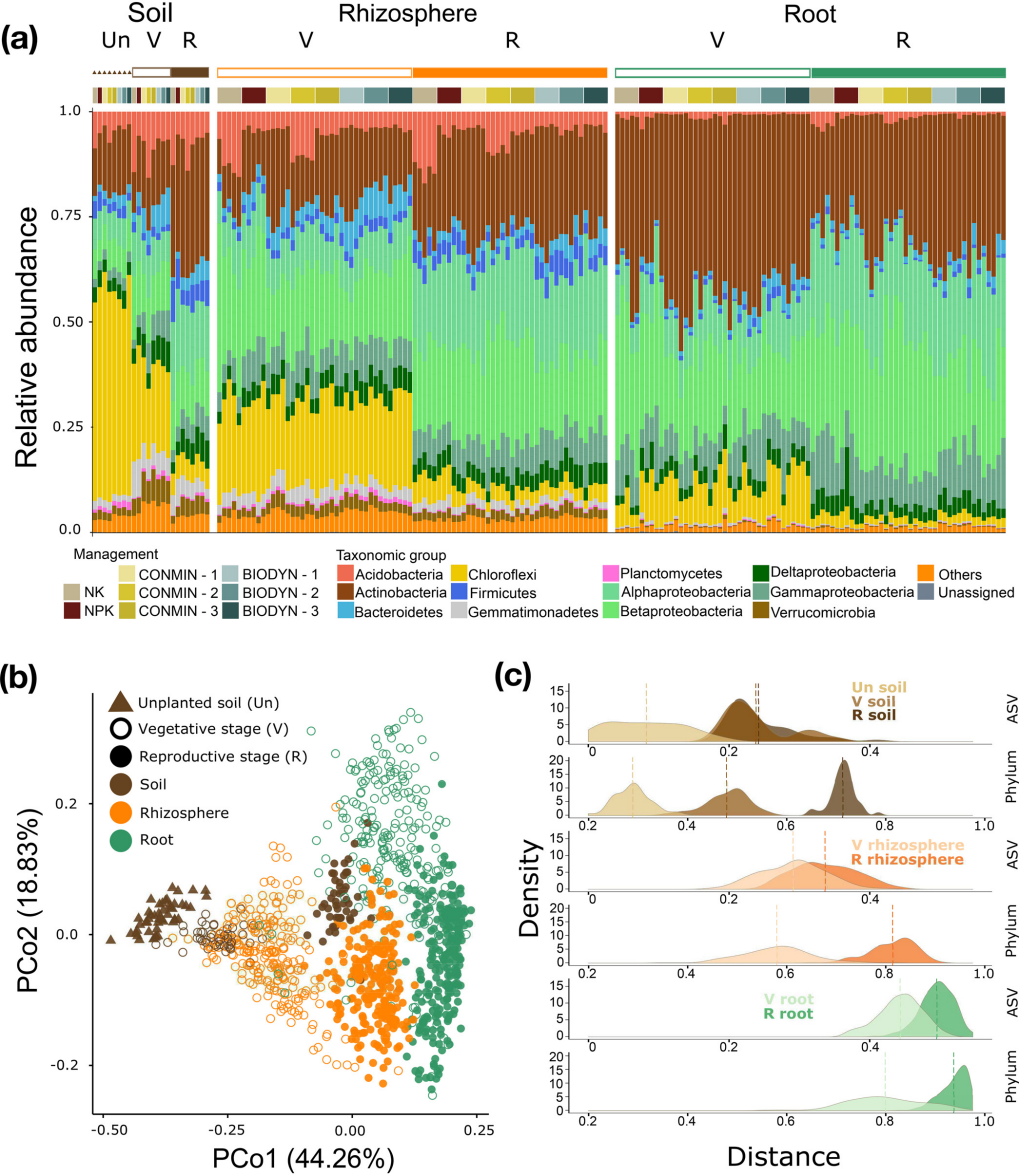
Plant growth phase shapes the bacterial community at the phylum level. (a) Relative abundances (RAs) of the 12 most abundant bacterial taxonomic groups. The taxonomic group “Others” gathers bacterial phyla with <0.1% RAs. (b) PCoA of Bray-Curtis dissimilarity based on the RA of each phylum between communities (*n* = 1,104). (c) Distribution of Euclidean distances between Bray-Curtis dissimilarities of each sample to the initial (unplanted soil before sowing) and final (reproductive root) communities. Comparison of bacterial communities was done at the ASV and phylum levels. The vertical line indicates the average distance of the corresponding condition.

To identify the principles governing community differentiation patterns, we compared the community profiles and diversities at different taxonomic levels (Fig. 3). Examination of the aRA values of each bacterial phylum demonstrated an unexpected convergence over time of microbial communities in the root microbiota, rhizosphere, and planted soil (Fig. 3a). In soil samples, aRAs of *Chloroflexi* and *Planctomycetes* were decreased over time, accounting for the main difference between the microbial biota of unplanted and planted soil. A significant enrichment of four taxonomic groups, namely, *Actinobacteria*, *Alphaproteobacteria*, *Betaproteobacteria*, and *Gammaproteobacteria*, was identified in root samples compared to other compartments, independent of plant growth (*P < *0.001). Moreover, a progressive decrease in the aRAs of *Acidobacteria*, *Bacteroidetes*, *Chloroflexi*, Deltaproteobacteria, *Firmicutes*, *Gemmatimonadetes*, *Planctomycetes*, and *Verrucomicrobia* was observed in a gradient from soil to rhizosphere to root samples, from the vegetative to the reproductive stage. In soil and rhizosphere, we also observed that *Acidobacteria* were significantly (*P < *0.001) enriched in the plots with the lowest pH values (NK and CONMIN-2) compared to the other plots under the same soil management, contributing to the higher similarities between those bacterial communities observed previously (Fig. 1a; see also Appendix 1 at the URL mentioned above).

In addition, a beta-diversity analysis was performed at the phylum level (Fig. 3b). As previously shown for bacteria (Fig. 1b) that at the amplicon sequence variant (ASV) level, the dissimilarities between samples were largely due to compartment. At the phylum level, however, samples from different growth stages within each compartment were separated. In particular, the later the soil and rhizosphere communities were harvested, the more these communities were similar to the root microbiota. To quantify the observed effect of the plant on the community structure across compartments at different growth stages, we defined initial and final bacterial communities (unplanted soil before sowing and reproductive phase of the root, respectively). Next, we calculated the Bray-Curtis dissimilarities (BCs) between each sample and both the initial and final communities. Euclidean distances between samples along these two dimensions showed a clear separation between stages only at the phylum level, consistent with what we observed from the principal-coordinate analysis (PCoA) (Fig. 1b and Fig. 3b). For instance, the distance distributions of soil samples from the vegetative and reproductive stages were almost overlapping at the ASV level (0.0037 average distance difference [nonsignificant *P* value]) but were significantly separated (0.23 [*P < *0.001]) at the phylum level.

The structure of fungal communities was shaped by the compartment and the plant growth stage, which is highlighted by the significant (*P < *0.001) enrichment of Glomeromycota in the root (Fig. S4a). The RA of this phylum decreased over the course of plant growth in the rhizosphere and root (*P < *0.001). We also performed beta-diversity analysis at the phylum level for fungal communities (Fig. S4b), which revealed an influence of compartment and host growth stage that is similar to that at the ASV level (Fig. 1b). This pattern was further confirmed by the distance distributions (Fig. S4c). For rhizosphere samples, the average distances between the vegetative and reproductive samples at the ASV and phylum levels were similar (0.16 and 0.15, respectively). For root samples, a stronger separation at the ASV level than at the phylum level was observed (0.15 and 0.048, respectively [*P < *0.001]). Taken together, ASV- and phylum-level distance distributions differ significantly for bacteria but not fungi.

10.1128/mBio.02584-21.5 FIG S4 Compartment and plant growth phase affect the fungal community similarly at the ASV and phylum levels. (a) RA values of the 15 most abundant fungal phyla. The taxonomic group “Others” gathers phyla with <0.1% RAs. (b) PCoA of Bray-Curtis dissimilarities based on the RA of each phylum between communities. (c) Comparison of fungal communities at the ASV and phylum levels. “Distance” on the *x* axis indicates the Euclidean distance between the Bray-Curtis dissimilarity of each sample and those of the initial (unplanted soil) and final (reproductive root) condition communities. The vertical line indicates the average distance of the corresponding condition. Un, unplanted soil before sowing; V, vegetative soil; R, reproductive soil. Download FIG S4, EPS file, 1.1 MB. Copyright © 2022 Bourceret et al. 2022 Bourceret et al. https://creativecommons.org/licenses/by/4.0/ This content is distributed under the terms of the Creative Commons Attribution 4.0 International license.

### Plant growth phase is a major driver of both root metabolism and root microbiota dynamics.

**FIG 4:**
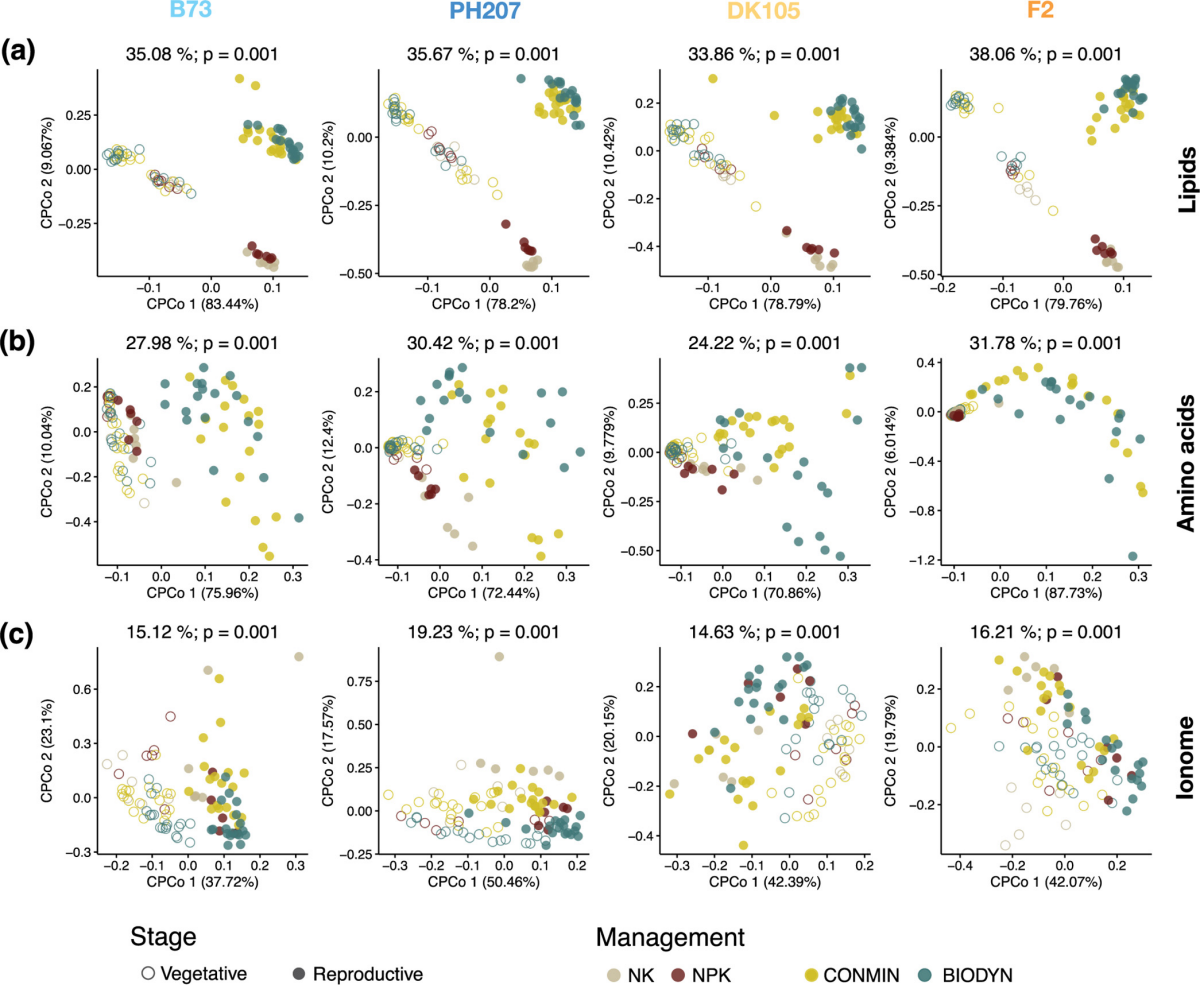
Root metabolites and total element compositions are affected by plant growth phase. A constrained PCoA (CPCoA) based on Euclidean distances between samples regarding the root lipid (a), amino acid (b), and ionomic (c) profiles of the four inbred lines was performed. The PCoA was constrained by soil management and plant growth phase for four inbred lines (59 lipid compounds were analyzed [*n* = 361], 15 amino acid compounds were analyzed [*n* = 376], and 20 total elements were analyzed [*n* = 384]). Each single point represents one analyzed root sample.

To assess the temporal dynamics of plant metabolism under different soil management regimes, we characterized the root metabolome and ionome of wild-type plants at the vegetative and reproductive growth stages, respectively (Fig. 4). Of all examined metabolite classes, the profile of root lipids was the most strongly affected by the plant growth phase, accounting for 34 to 38% of the variance between samples of the four tested maize inbred lines (Fig. 4a). When analyzing the individual lipids, we observed a general increase at the reproductive stage compared to the vegetative stage for most classes, irrespective of soil management and genetic background (Fig. S5). This response was more evident when comparing the soil managements CONMIN and BYODYN at both developmental stages. In this case, the levels of most of the annotated monogalactosyldiacylglycerols (MGDGs), digalactosyldiacylglycerols (DGDGs), and triacylglycerols (TAGs) were substantially increased at the reproductive stage. An effect of soil management was also evident, especially at the vegetative stage. For instance, the levels of most of the MGDGs, DGDGs, and TAGs were reduced under CONMIN and BIODYN compared to NK and NPK. Some exceptions were MGDGs 34.3, 36.5, and 36.6 and phosphatidylcholine (PC) 36.6, which showed an opposing response. At the reproductive stage, phosphatidylserine (PS), sulfoquinovosyldiacylglycerol (SQDG), and diacylglycerol (DAG) lipids also decreased under CONMIN and BIODYN compared to NK and NPK. We found similar but lower overall profile changes for amino acids (24 to 32% of the variance for different inbred lines) (Fig. 4b) and the ionome (15 to 19% of the variance for different inbred lines) (Fig. 4c), with plant growth phase again being the main explanatory factor. We also determined the sugar composition of roots, but the corresponding metabolite profiles were limited to the DEMO field (Fig. S6). These results confirmed that host growth phase is the most important explanatory variable for the root metabolite dynamics of all metabolite classes tested.

10.1128/mBio.02584-21.6 FIG S5 Plant growth stage shapes the root lipid profile. A heat map of the 59 lipid compounds in root samples from two growth stages, four soil managements, and four inbred lines is shown. Averages of log-transformed and range-normalized lipid profiles under each condition are shown. Download FIG S5, EPS file, 0.3 MB. Copyright © 2022 Bourceret et al. 2022 Bourceret et al. https://creativecommons.org/licenses/by/4.0/ This content is distributed under the terms of the Creative Commons Attribution 4.0 International license.

10.1128/mBio.02584-21.7 FIG S6 Root sugar composition is affected by the plant growth stage. CPCoA (∼management × stage) was based on Euclidean distances of the root sugar composition (sucrose, fructose, and glucose) for the four inbred maize lines grown in the DEMO field (NK and NPK) (*n* = 93). Download FIG S6, EPS file, 0.1 MB. Copyright © 2022 Bourceret et al. 2022 Bourceret et al. https://creativecommons.org/licenses/by/4.0/ This content is distributed under the terms of the Creative Commons Attribution 4.0 International license.

We then tested for covaried microbial taxa and root lipids and identified a group of predictive taxa, including both stable (widespread and persistent from the vegetative to the reproductive stages) and dynamic OTUs (Fig. S7). Approximately half of the most significantly covaried taxa (OTUs with the top 25 maximum mean squared error values) were stable bacterial (12 OTUs, 10 from *Proteobacteria* and 2 from *Actinobacteria*, out of 26 stable root OTUs in Fig. 2a) and fungal (10 OTUs, 9 from Ascomycota and 1 from Glomeromycota, out of 27 stable root OTUs in Fig. 2c) taxa in the root (Fig. S7a and b; see also Tables S2 to S7 at https://github.com/Guan06/Bourceret_and_Guan_et_al_2022). We identified the microbial taxa that were significantly (*P < *0.05) and strongly (|*r*| > 0.5) correlated with lipids (Fig. S7c). Predictive *Proteobacteria* taxa were mainly found to be positively correlated with lipid profiling. *Chloroflexi*, a phylum that was decreased during plant growth in the root (Fig. 3a), were negatively correlated with most of the lipids. For fungal predictive OTUs, except for one Ascomycota OTU, most taxa were negatively correlated with lipids (Fig. S7c). Taken together, our data indicate that both the root metabolome and the root microbiota respond most strongly to plant growth phase and that there is a correlation between stable and dynamic root microbial taxa and root lipids during plant growth.

10.1128/mBio.02584-21.8 FIG S7 The root-associated microbiota covaries with the root lipid profile over plant growth. (a and b) The most discriminatory (with the highest mean squared error calculated by random forest) (*n* = 25) bacterial (a) and fungal (b) OTUs for lipid in root and their phylum affiliations. Salmon corresponds to the persistent OTUs in the root shown in Fig. 2, and gray indicates nonpersistent OTUs. (c) Network of the most predictive bacterial and fungal OTUs and lipid compounds. Strong (absolute value > 0.5) and significant (*P* < 0.05) Spearman correlations were kept as edges (*n* = 25 OTUs for both microbial kingdoms). The circle of each node shows the ratio of the average RAs of the corresponding OTU or lipid between the vegetative stage (light green) and reproductive stage (dark green) roots. Download FIG S7, PDF file, 0.2 MB. Copyright © 2022 Bourceret et al. 2022 Bourceret et al. https://creativecommons.org/licenses/by/4.0/ This content is distributed under the terms of the Creative Commons Attribution 4.0 International license.

### Genotype-dependent responses of root microbiota, metabolism, and plant biomass to soil P availability.

To assess the responses of the host plant, root metabolism, and root microbiota to nutrient availability, we introduced a *pht1*;*6* P transporter mutant of B73, whose transport of bioavailable P from AMF to the plant host is impaired, thereby compromising the establishment of maize-AMF symbiosis (40). We first compared the degrees of mycorrhizal root colonization and the RAs of Glomeromycota in root samples of the wild type and mutant under different managements and at different growth stages (Fig. S8). As expected, in the *pht1*;*6* mutant, we found significant decreases in mycorrhizal colonization (based on microscopy) and RAs (based on amplicon sequencing) of Glomeromycota at the vegetative stage (Fig. S8a). Surprisingly, at the reproductive stage of wild-type plants, the mycorrhizal colonization ratio, which was calculated only in fine roots by microscopy, increased (Fig. S8a), while the average RA of Glomeromycota in the entire root system decreased (Fig. S8b).

10.1128/mBio.02584-21.9 FIG S8 Relative abundance of Glomeromycota and mycorrhizal colonization in roots are influenced by soil management, the P transporter Pht1;6, and plant growth stage. (a) Degrees of colonization of mycorrhizal fungi over the course of plant growth in the B73 wild-type line and the *pht1*;*6* mutant in the four soil managements. Colonization was estimated by microscopic counting on fine roots. (b) RAs of Glomeromycota in the B73 wild-type line and the P transporter mutant line over the course of plant growth. A Wilcoxon test with FDR correction was used for statistical analysis (*P < *0.05) (*n* = 6 for NK and NPK; *n* = 18 for CONMIN and BIODYN). Lowercase letters indicate significant differences between managements at the two growth stages within each genotype. Capital letters indicate significant differences between two genotypes, and asterisks indicate significant differences between two growth stages, including all managements. Download FIG S8, EPS file, 0.2 MB. Copyright © 2022 Bourceret et al. 2022 Bourceret et al. https://creativecommons.org/licenses/by/4.0/ This content is distributed under the terms of the Creative Commons Attribution 4.0 International license.

**FIG 5:**
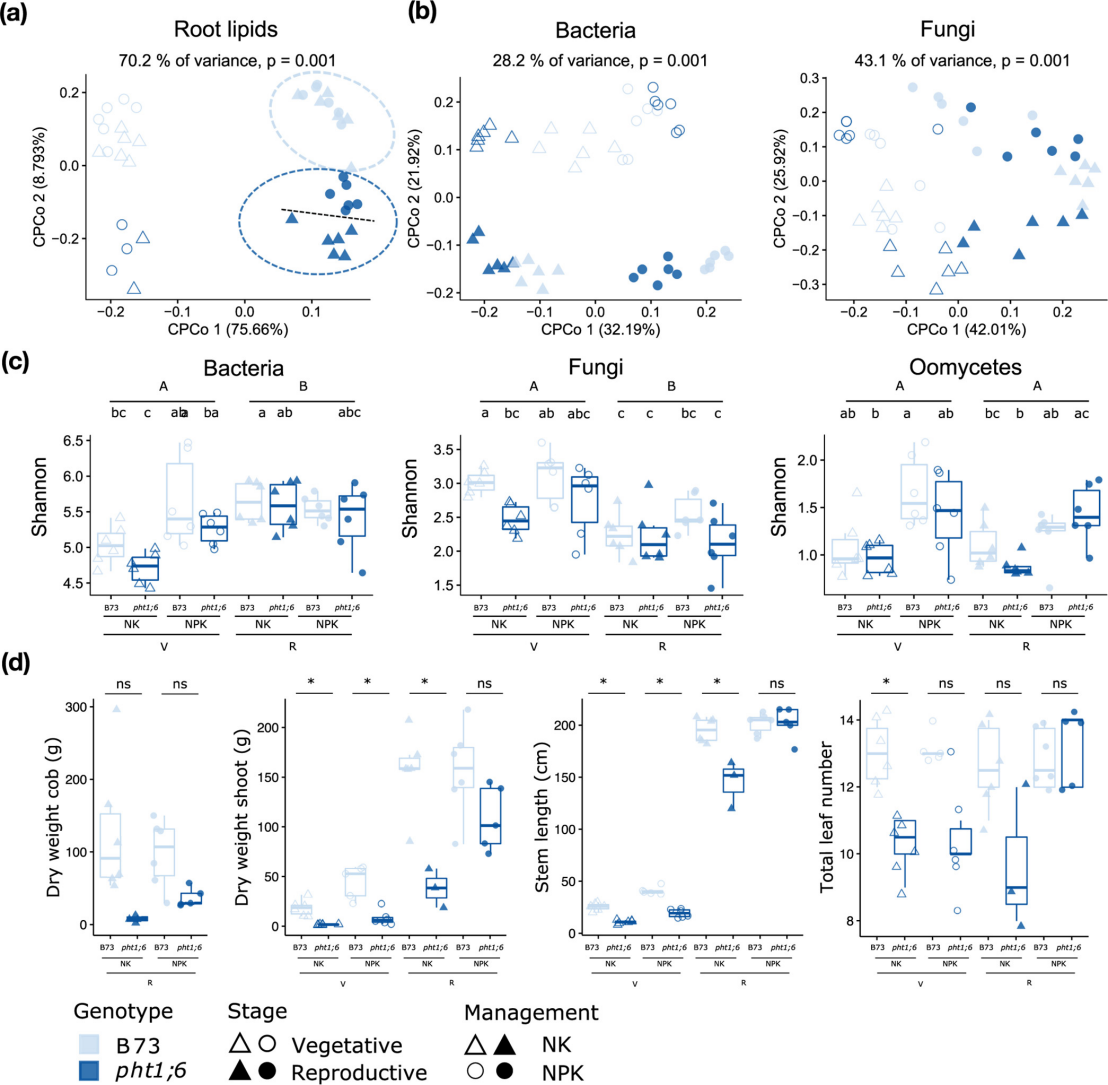
Genotype-dependent effect of phosphate depletion on root lipids, microbiota, and plant biomass. (a) CPCoA based on the Euclidean distance of the root lipid profiles of the wild type (WT) and the P transporter-defective line *pht1*;*6* at both growth stages (71 lipid compounds were analyzed [*n* = 41] [data can be found at GitHub]). (b) CPCoA based on Bray-Curtis dissimilarity of the root bacterial and fungal communities (*n* = 48 for bacterial and fungal communities) and constrained by plant genotype, plant growth stage, and soil management (depletion/amendment of P on soil). (c) Effects of genotype, plant growth stage, and P fertilization on microbial community alpha-diversity (*n* = 6 per plot) (V, vegetative stage; R, reproductive stage). (d) Effects of genotype, plant growth stage, and P fertilization on four plant biomass parameters (*n* = 43). A Wilcoxon test followed by FDR correction was used for statistical analysis (*P < *0.05). Capital letters indicate significant differences between growth stages; lowercase letters indicate significant differences between genotypes in specific soil management, within each growth stage. ns, not significant.

Afterward, to specify the P effect, we examined root lipid, root microbiota, and plant phenotypic data of samples derived from wild-type B73 and mutant *pht1*;*6* plants grown in NK and NPK soil managements only (Fig. 5). Root lipid compositions were mainly influenced by growth stage (1st axis) and plant genotype (2nd axis) (Fig. 5a; see also Tables S2 to S7 at the URL mentioned above). In addition, at the reproductive stage, NPK and NK managements resulted in different lipid profiles in *pht1*;*6* roots (Fig. 5a), indicating a potential plant growth phase-specific link between AMF symbiosis, root lipid status, and soil P availability. The soil P availability-dependent change in the root lipid profiles was paralleled by alterations in root-associated bacterial and fungal communities (Fig. 5b). The primary driver of the bacterial community shift was P management (1st axis, 13.39%) (see Tables S2 to S7 at the URL mentioned above), whereas for fungi, samples clustered mainly according to plant growth stage (25.54%) (see Tables S2 to S7 at the URL mentioned above). We also detected a trend of lower bacterial and fungal alpha-diversities in *pht1*;*6* roots than in wild-type roots and in NK-grown plants than in NPK-grown plants at the vegetative stage (Fig. 5c), indicating that AMF and soil P deficiency have a broad effect on bacterial and fungal root-associated assemblages. This effect was undetectable at the reproductive growth stage, suggesting compensatory changes in the maize root microbiota over time. Consistent with this interpretation, we found that the marked differences in the tested plant performance parameters in *pht1*;*6* compared to wild-type plants, seen at the vegetative growth phase, were mostly compensated for at the reproductive growth stage (Fig. 5d).

## DISCUSSION

### Soil physicochemical properties drive soil- and plant-associated microbial community shifts across different microbial kingdoms.

Our results showed that in a long-term agricultural system, the soil biota composition was strongly impacted by abiotic factors determined by geographical location, soil management, and plot location. These factors were responsible for the variability in soil physicochemical properties and differentially shaped the communities of bacteria, fungi, and oomycetes in the rhizosphere and root compartments.

We found a slightly higher alpha-diversity for the bacterial communities in DOK (see Fig. S3a in the supplemental material), possibly linked to differences in the size distributions of soil aggregation between the DEMO and DOK fields. The particle size influences the oxygen concentration and moisture availability of the soil microenvironment (41) and therefore will affect all microbes living there. Moreover, differences in climatic conditions, such as rainfall and air temperature, likely contribute to the effect of geographical location. Consistent with recently characterized natural populations of *A. thaliana* and cultivated and native *Agave* species (42, 43), we observed that the community of filamentous eukaryotes (fungi and oomycetes) is more strongly influenced by geographical location than the bacterial community (Fig. 1; Fig. S2 and S3b). However, because the two fields were not managed identically, this confounds the effects of geographical and management factors.

Through modification of the edaphic parameters within a given field, soil management also modifies the soil biota (13, 19, 44). These effects are mainly apparent with respect to community structure and are not as strong with regard to alpha-diversity (45). Consistent with this, mineral NK and NPK fertilizations in the DEMO field were associated with a distinguishable soil biota (Fig. 1; Fig. S2 and S8), probably depending on the soil P level (readily available and aqua regia-extractable P). However, the impact of other soil factors cannot be excluded (e.g., POXC: labile organic carbon or pH). The nonsignificant differences in bacterial and fungal Shannon indices between NK and NPK (Fig. S3a) indicate that the alpha-diversity of the microbial soil biota is largely resilient to long-term P nutrient supplementation, as previously suggested (46). In the DOK field, comparison of CONMIN and BIODYN soil properties featured higher pH and elevated contents of organic carbon (C_org_), POXC, total N, C/N, and available P in the organic treatment (Table S1b). Previous studies in the same field showed a positive effect of farmyard manure (FYM), used in BIODYN management, on microbial abundance and activity (47, 48) and community composition (44). In line with the concept of *r-* and *K-*selection theory applied to soil microbial communities, in which the selection of bacterial communities is driven by nutrient availability and their ecological strategies (49), we found that high-growth-rate microbes such as some *Bacteroidetes* (*r*-strategists) were promoted by the abundant nutrients provided by FYM in BIODYN. This might be due to their ability to degrade complex organic compounds (50).

10.1128/mBio.02584-21.10 TABLE S1 Four different soil managements, NK, NPK, CONMIN, and BIODYN, were applied on the DEMO (fertilization demonstration experiment) (Reckenholz) and DOK (dynamic, organic, and conventional managements) (Therwil) long-term fields, and the corresponding soil parameters were measured on unplanted soil before sowing. (a) N_tot_, total nitrogen; N_org_, organic nitrogen; N_min_, mineralizable nitrogen; NK, N and K fertilization; NPK, N, P, and K fertilization; CON, CONMIN (conventional mineral fertilization); BIO, BIODYN (biodynamic). In the “Input” section, the concentration of complex compounds amended on soil is specified, and the resulting amounts of P and K are symbolized by the arrow. (b) Measurements were performed on the DEMO and DOK fields subjected to four different soil managements (NK, NPK, CONMIN, and BIODYN). pH H_2_O, pH in aqueous solution; pH CaCl_2_, pH in salt solution; C_org_, organic carbon; N, total nitrogen; C/N, carbon/nitrogen ratio; POXC, labile organic carbon, sand content, silt content, and clay content; LM, large macroaggregates (>2,000 μm); SM, small macroaggregates (250 to 2,000 μm); μA, microaggregates (53 to 250 μm); S+C, silt and clay fraction (<53 μm); CEC, cation exchange capacity; BS, base saturation; Mno, Mn(III) oxide content; Feo, short-range ordered Fe(III) oxide content; Fed, total FeIII oxide content; Mg, magnesium; K, potassium; Ca, calcium; P, phosphorus; Mn, manganese; Fe, iron; Cu, copper; Zn, zinc; Na, sodium; Mo, molybdenum; Al, aluminum; KW, aqua regia-extractable elements; plant-available nutrients, CO_2_-saturated water-extracted nutrients. Means and standard deviations are shown (*n* = 6 for C_org_, N, C/N, and POXC; *n* = 12 for LM, SM, and μA; and *n* = 4 for S+C). Download Table S1, PDF file, 0.2 MB. Copyright © 2022 Bourceret et al. 2022 Bourceret et al. https://creativecommons.org/licenses/by/4.0/ This content is distributed under the terms of the Creative Commons Attribution 4.0 International license.

At a local scale within each management, some soil properties were highly variable between different plots, and bacteria were more impacted than filamentous eukaryotes by this heterogeneity at the plot scale (Fig. 1a; Fig. S2 and Table S1b). The sensitivity of bacteria to pH, which has also been reported previously (51, 52), and to clay highlights the importance of edaphic parameters for the bacterial community structure. A shift of around 1 pH unit led to an enrichment of *Acidobacteria* in the NK and CONMIN-2 plots, regardless of their contrasting managements and field locations (Fig. 3a).

Horizontal gene transfers from fungi to oomycetes converge within the radiation of oomycetes capable of colonizing plant tissues and are associated with the transition to their predominant phytopathogenic lifestyle (53). We observed a strong effect of soil management on the root-associated microbiota (Fig. 1), notably for oomycetes, suggesting that adapted agricultural practices can be instrumental in reducing damage caused by these ubiquitous phytopathogens. We found that the interaction between host genotype and management also explained a large degree of the variance in communities. This interaction suggests that plant microbiota composition is not only impacted by soil management but also determined by the degree to which specific plant genotypes are adapted to specific environments.

### Temporal changes in maize root-associated bacterial communities covary with root metabolite dynamics.

We have shown that the effect of the soil management and plant genotype interaction on shaping the root microbiota was relatively stable between the vegetative and reproductive growth stages for the three microbial kingdoms (Fig. 1; see also Tables S2 to S7 at https://github.com/Guan06/Bourceret_and_Guan_et_al_2022). Time course experiments in rice have shown the rapid acquisition and stable taxonomic structure of the bacterial root microbiota within 14 days after transplantation from sterile medium to soil (19). A subsequent study comparing the root microbiota of field-grown rice over the course of three growing seasons, including four cultivars, revealed changes in bacterial composition from the vegetative to the reproductive stages and identified predictive microbiota reflecting plant age (20). Plant age-dependent variation in rhizosphere bacterial communities of field-grown maize over a 20-week period with weekly sampling, spanning the vegetative and reproductive growth phases, showed gradual rather than two-stage community shifts (21). In light of these findings, the distinctive microbial profiles detected in our study at the vegetative and reproductive growth stages for the rhizosphere and root compartments are likely two snapshots of a gradual maize root-associated microbiota dynamic over time. In field-grown maize with weekly samplings, a core of seven bacterial OTUs shared in all rhizosphere samples was identified (21). In this study, we found 15 stable bacterial OTUs, shared between the rhizosphere and root compartments as well as between the vegetative and reproductive growth phases, representing approximately one-third of the root microbiota (Fig. 2). A comparison of the seven common rhizosphere bacterial OTUs found in U.S. fields with the 15 OTUs identified here in European field-grown maize shows an overlap at the rank of family (5/7 U.S. and 7/15 European OTUs with shared family assignment, respectively) (see Tables S2 to S7 at the URL mentioned above), including *Bradyrhizobiaceae*, *Comamonadaceae*, *Pseudomonadaceae*, and *Sinobacteraceae*. This overlap indicates the existence of stable community members that define the maize bacterial root microbiota across two continents despite different host genotypes, soil managements, climates, and soil types.

The gradual shift in microbiota composition over maize growth (21) and the uncoupling of genetically determined flowering time in *A. alpina* from soil residence time-dependent changes in its root microbiota (32) make it unlikely that genetically determined vegetative and reproductive growth stages control the temporal dynamics of the bacterial root microbiota. Instead, the dynamics of root metabolites over the growing season (Fig. 4) could drive bacterial succession in a “shell” surrounding a stable microbiota core, enabled by bacterial immigration from the soil biome. According to our study, this dynamic shell comprises up to two-thirds of the bacterial root microbiota. This model is supported by a study in monocotyledonous *A. barbata*, in which it was shown that chemical succession in root exudation over the growing season explains part of the bacterial community assembly and dynamics in the rhizosphere (31). However, we found that the majority of the fungal root community is stable over the tested maize growth phases, indicating that the root-associated fungi are less responsive to changes in root metabolites.

At a high taxonomic level, we found a shift in the bacterial community toward a structure resembling that of later-stage roots in all of the compartments, including planted soil (Fig. 3). The large expansion of the maize root system over time in the field might extend the spatial chemical gradient from the plant to the soil, inducing this shift. This plant footprint was observed only at the phylum level, indicating that the root shapes bacterial communities based on their conserved metabolic (functional) potential, independently of intraspecies (ASV-level) diversity (54). We propose that this plant footprint on the bacterial communities is partially driven by the dynamics of root metabolites over time that spread via exudation beyond the rhizosphere into the soil. BX and its stable degradation product MBOA are candidate maize root-secreted chemicals that could contribute to this mechanism (37–39). In contrast, for fungal communities, we found similar assembly patterns at the ASV and phylum levels over the growing season across all tested compartments, corroborating that fungi are more resistant to changes in root metabolism. It remains to be tested whether this difference reflects fundamentally different preferences of bacteria and fungi for root-derived nutrients and/or sensitivity to phytochemicals.

### Soil P availability induces changes in root metabolism, plant performance, and root microbiota.

By comparing wild-type and P transporter *pht1*;*6* mutant plants, we showed a potential plant growth phase-specific link between AMF symbiosis, root lipid status, and soil P availability (Fig. 5). In AMF-deficient *pht1*;*6* plants during reproductive maize growth, root lipid status varied the most in response to P availability. The establishment of AMF symbiosis in roots is promoted under P-limiting conditions (NK), and during symbiosis, the fungi can provide the dominant route for plant P supply (40, 55). In return, host-derived lipids are a major source of organic carbon delivered to fatty acid auxotrophic AMF for fungal growth (56–59).

We found that the root lipid status between wild-type and AMF-deficient *pht1*;*6* plants differed more strongly under NK than under NPK conditions, which is likely linked to perturbed cross-kingdom lipid transfer from the host to AMF in the mutant roots. We also observed that the performance of *pht1*;*6* mutants was reduced compared to that of the wild type, as previously shown (40, 60), and more severely at the vegetative growth phase. These performance differences were partially compensated for at the reproductive growth phase under P-sufficient conditions (NPK). The lack of full growth compensation points to additional AMF-independent functions of the Pht1;6 transporter for maize growth and/or additional beneficial AMF symbiosis activities independent of P supply to the host. Additionally, we found that contrasting P availability induced changes in the bacterial root microbiota that exceeded host growth phase-dependent microbiota variation in both wild-type and *pht1*;*6* plants. This indicates that the bacterial root microbiota actively responds to the host’s P status, as shown in *A. thaliana* by the direct integration of P stress and plant immune responses (17, 61), thereby likely contributing to overall host performance.

This work provides insights into the spatiotemporal dynamics of the maize root-associated microbiota by revealing an inverse stable-to-dynamic ratio between root-associated bacterial and fungal communities over the growing season. The future development of a gnotobiotic maize growth system and defined (synthetic) microbial communities should allow direct tests of whether changes in root metabolites drive succession in the dynamic shell of the bacterial root community, whereas stable bacterial and fungal root microbiota members have adapted to host metabolite alterations.

## MATERIALS AND METHODS

### Experimental design.

The experiment was performed on two long-term fields located in Switzerland, DEMO (fertilization demonstration experiment) (Agroscope research station, Zürich-Reckenholz [47°25′31″N, 8°30′59″E; mean annual temperature, 9.4°C; mean annual precipitation, 1,031 mm], established in 1987) and DOK (dynamic, organic, and conventional managements) (Therwil [47°30′09″N, 7°32′21″E; mean annual temperature, 10.5°C; mean annual precipitation, 842 mm], established in 1978) (44), where different soil managements (encompassing a combination of soil fertilization and agricultural practices) were applied (see Text S1, Table S1a, and Fig. S1 in the supplemental material). The soil type at DEMO is Gleyic Cambisol, and that at DOK is Haplic Luvisol, according to the FAO (62). In the DEMO field, NPK (nitrogen, phosphate, and potassium) and NK (nitrogen and potassium) managements were compared. In DOK, biodynamic mixed (BIODYN) and conventional solely mineral-fertilized (CONMIN) managements, with three replicate plots dispersed within the field (locations 1, 2, and 3) (Fig. S1), were used.

10.1128/mBio.02584-21.2 FIG S1 Experimental design. Five different maize genotypes, including four inbred lines (B73, DK105, PH207, and F2) and one phosphate transporter mutant line (*pht1*;*6*), compromised in phosphate transport from fungi to the plant, were planted in two long-term experimental fields, DEMO (fertilization demonstration experiment) (Reckenholz) and DOK (dynamic, organic, and conventional managements) (Therwil), in Switzerland. Two soil managements per field were tested. NK and NPK soil fertilizations were practiced in DEMO (one plot per management), and CONMIN and BIODYN were used in DOK (three plots per management). Six unplanted soil samples per plot were collected before sowing. Later, at the vegetative and reproductive stages of plant growth, six plants per genotype were harvested. For each plant, rhizosphere and root samples were collected. Additional bulk soil was sampled from each planted plot (*n* = 3) at both stages. Download FIG S1, EPS file, 0.5 MB. Copyright © 2022 Bourceret et al. 2022 Bourceret et al. https://creativecommons.org/licenses/by/4.0/ This content is distributed under the terms of the Creative Commons Attribution 4.0 International license.

Within each plot, five maize genotypes were planted: inbred lines B73 and PH207 from genetic pool Dent and DK105 and F2 from Flint in addition to mutant line *pht1*;*6* derived from B73 with a mutation in the mycorrhiza-specific Pht1;6 P transporter gene (40). The examined genetic pools correspond to a classification that considers the structure of the grain and differentiation in traits such as flowering time and cold tolerance (63). For each genotype, six rows (with a 75-cm distance) of five plants each (15-cm distance) were distributed per plot (*n* = 30). Plants were grown and harvested at two time points, 7 weeks (July 2017) and 15 weeks (September 2017) after sowing, corresponding approximately to the vegetative (V6) and reproductive (R2) stages of plant development, respectively (64). The number of samples collected under each condition is indicated in Text S1.

10.1128/mBio.02584-21.1 TEXT S1 Supplemental materials and methods. Download Text S1, DOCX file, 0.04 MB. Copyright © 2022 Bourceret et al. 2022 Bourceret et al. https://creativecommons.org/licenses/by/4.0/ This content is distributed under the terms of the Creative Commons Attribution 4.0 International license.

### Soil and plant sampling.

For soil property measurements, six disturbed soil samples (2 kg) from each unplanted plot were collected before sowing (*n* = 48). Topsoil samples were collected from 5 to 20 cm below the surface along a linear transect to assess the effect of spatial variability within each plot (Text S1), and 38 soil parameters were measured (Table S1b). For soil microbiota analysis, a subsample of 50 g from each soil sample was flash-frozen in liquid nitrogen and stored at −80°C until analysis.

For each genotype, six healthy plants were harvested at each time point. To prevent any border or cross-genotype effect, plants in the middle of the plot (harvesting area) with at least one plant from the border or a different genotype were harvested, preferentially. After removing most of the attached soil, a representative sample of the root system (including different types of roots) was collected and flash-frozen in liquid nitrogen. Additionally, for each planted plot at each time point, 10 g of bulk soil (*n* = 3) was collected (−5 to −20 cm from the surface) between two rows of plants for microbiota analyses.

### Plant biomass and root metabolism measurements.

For each harvested plant, the stem length, leaf number, and developmental status were determined. After measuring the fresh weight, the shoot was cut into pieces, dried at 65°C at Reckenholz over several days, and ground, and the dry weight was then measured. The cobs of each plant were weighed similarly. For metabolome (lipid, sugar, and free amino acids) and total elemental compositions, frozen washed root tissues were homogenized to a fine powder, and 50 to 100 mg was used (details are described in Text S1). Fine roots of maize plants were harvested at the vegetative and reproductive stages in 70% ethanol (EtOH), and mycorrhizal colonization was assessed using ink staining (Text S1).

### Soil and root microbial community profiling.

The collected root samples were fractionated into rhizosphere and endosphere (referred to as root here) fractions in the laboratory using a protocol adapted from the one described previously by Bulgarelli et al. (65). Total genomic DNA was extracted from bulk soil, rhizosphere, and root samples from at least 250 mg of material using the FastDNA Spin kit for soil (MP Biomedicals, Solon, OH, USA). The concentration of DNA samples was measured by fluorescence (Quant-IT PicoGreen; Invitrogen, OR, USA). DNA samples diluted to 3.5 ng/μL were amplified in triplicate in a two-step PCR using specific primer sets for the profiling of bacterial (V5-V7 region of 16S rRNA) (primer pair 799F/1192R), fungal (internal transcribed spacer 2 [ITS2]) (fITS7/ITS4), and oomycetal (ITS1) (ITS1-O/5.8s-O-rev) communities, as described previously by Robbins et al. (46) (Text S1). Illumina sequencing was performed at the Cologne Center for Genomics (CCG) using the MiSeq platform and custom sequencing primers (see Tables S2 to S7 at https://github.com/Guan06/Bourceret_and_Guan_et_al_2022).

### Amplicon sequencing data processing and microbial community diversity analysis.

We applied a pipeline based on DADA2 (v1.12.1) (66) for data processing and the generation of an amplicon sequence variant (ASV) table. The taxonomy of ASVs was assigned by the naive Bayesian classifier (67), using SILVA (v132), UNITE (release 02.02.2019), and an in-house database described previously by Durán et al. (9) for bacteria, fungi, and oomycetes, respectively. ASV tables were rarefied to depths of 1,000, 10,000, and 2,000 for bacteria, fungi, and oomycetes, respectively. The Shannon index of alpha-diversity was then calculated as the average value from 999 independent rarefactions. The average was then computed as the alpha-diversity of communities. The Bray-Curtis dissimilarity (BC) between samples was calculated based on rarefied ASV tables for beta-diversity analysis at the ASV level. Permutational multivariate analysis of variance (PERMANOVA) was performed with the adonis() function in the R package vegan (68). Bacterial OTUs were clustered with an identity of 97% from ASVs.

### Data availability.

The raw sequencing data described in the manuscript were uploaded to the European Nucleotide Archive under accession number PRJEB44300. Both the modified DADA2 pipeline for data processing (https://github.com/Guan06/DADA2_pipeline) and scripts as well as clean data for visualization (https://github.com/Guan06/Bourceret_and_Guan_et_al_2022) are available at GitHub.

## References

[1] Berendsen RL, Pieterse CMJ, Bakker PAHM. 2012. The rhizosphere microbiome and plant health. Trends Plant Sci 17:478–486. doi:10.1016/j.tplants.2012.04.001. 22564542

[2] Hassani MA, Durán P, Hacquard S. 2018. Microbial interactions within the plant holobiont. Microbiome 6:58. doi:10.1186/s40168-018-0445-0. 29587885 PMC5870681

[3] Bulgarelli D, Schlaeppi K, Spaepen S, van Themaat EVL, Schulze-Lefert P. 2013. Structure and functions of the bacterial microbiota of plants. Annu Rev Plant Biol 64:807–838. doi:10.1146/annurev-arplant-050312-120106. 23373698

[4] Hacquard S, Garrido-Oter R, González A, Spaepen S, Ackermann G, Lebeis S, McHardy AC, Dangl JL, Knight R, Ley R, Schulze-Lefert P. 2015. Microbiota and host nutrition across plant and animal kingdoms. Cell Host Microbe 17:603–616. doi:10.1016/j.chom.2015.04.009. 25974302

[5] Bucher M. 2007. Functional biology of plant phosphate uptake at root and mycorrhiza interfaces. New Phytol 173:11–26. doi:10.1111/j.1469-8137.2006.01935.x. 17176390

[6] Pii Y, Mimmo T, Tomasi N, Terzano R, Cesco S, Crecchio C. 2015. Microbial interactions in the rhizosphere: beneficial influences of plant growth-promoting rhizobacteria on nutrient acquisition process. A review. Biol Fertil Soils 51:403–415. doi:10.1007/s00374-015-0996-1.

[7] Hiruma K, Gerlach N, Sacristán S, Nakano RT, Hacquard S, Kracher B, Neumann U, Ramírez D, Bucher M, O’Connell RJ, Schulze-Lefert P. 2016. Root endophyte Colletotrichum tofieldiae confers plant fitness benefits that are phosphate status dependent. Cell 165:464–474. doi:10.1016/j.cell.2016.02.028. 26997485 PMC4826447

[8] Harbort CJ, Hashimoto M, Inoue H, Niu Y, Guan R, Rombolà AD, Kopriva S, Voges MJEEE, Sattely ES, Garrido-Oter R, Schulze-Lefert P. 2020. Root-secreted coumarins and the microbiota interact to improve iron nutrition in Arabidopsis. Cell Host Microbe 28:825–837.e6. doi:10.1016/j.chom.2020.09.006. 33027611 PMC7738756

[9] Durán P, Thiergart T, Garrido-Oter R, Agler M, Kemen E, Schulze-Lefert P, Hacquard S. 2018. Microbial interkingdom interactions in roots promote Arabidopsis survival. Cell 175:973–983.e14. doi:10.1016/j.cell.2018.10.020. 30388454 PMC6218654

[10] Kamoun S, Furzer O, Jones JDG, Judelson HS, Ali GS, Dalio RJD, Roy SG, Schena L, Zambounis A, Panabières F, Cahill D, Ruocco M, Figueiredo A, Chen X-R, Hulvey J, Stam R, Lamour K, Gijzen M, Tyler BM, Grünwald NJ, Mukhtar MS, Tomé DFA, Tör M, Van Den Ackerveken G, McDowell J, Daayf F, Fry WE, Lindqvist-Kreuze H, Meijer HJG, Petre B, Ristaino J, Yoshida K, Birch PRJ, Govers F. 2015. The top 10 oomycete pathogens in molecular plant pathology. Mol Plant Pathol 16:413–434. doi:10.1111/mpp.12190. 25178392 PMC6638381

[11] Benhamou N, le Floch G, Vallance J, Gerbore J, Grizard D, Rey P. 2012. Pythium oligandrum: an example of opportunistic success. Microbiology (Reading) 158:2679–2694. doi:10.1099/mic.0.061457-0. 22977087

[12] Chowdhury SP, Babin D, Sandmann M, Jacquiod S, Sommermann L, Sørensen SJ, Fliessbach A, Mäder P, Geistlinger J, Smalla K, Rothballer M, Grosch R. 2019. Effect of long-term organic and mineral fertilization strategies on rhizosphere microbiota assemblage and performance of lettuce. Environ Microbiol 21:2426–2439. doi:10.1111/1462-2920.14631. 30990945 PMC6849853

[13] Schmidt JE, Kent AD, Brisson VL, Gaudin ACM. 2019. Agricultural management and plant selection interactively affect rhizosphere microbial community structure and nitrogen cycling. Microbiome 7:146. doi:10.1186/s40168-019-0756-9. 31699148 PMC6839119

[14] Banerjee S, Walder F, Büchi L, Meyer M, Held AY, Gattinger A, Keller T, Charles R, van der Heijden MGA. 2019. Agricultural intensification reduces microbial network complexity and the abundance of keystone taxa in roots. ISME J 13:1722–1736. doi:10.1038/s41396-019-0383-2. 30850707 PMC6591126

[15] Fließbach A, Mäder P, Oberholzer H-R, Gunst L. 2007. Soil organic matter and biological soil quality indicators after 21 years of organic and conventional farming. Agric Ecosyst Environ 118:273–284. doi:10.1016/j.agee.2006.05.022.

[16] Francioli D, Schulz E, Lentendu G, Wubet T, Buscot F, Reitz T. 2016. Mineral vs. organic amendments: microbial community structure, activity and abundance of agriculturally relevant microbes are driven by long-term fertilization strategies. Front Microbiol 7:1446. doi:10.3389/fmicb.2016.01446. 27683576 PMC5022044

[17] Castrillo G, Teixeira PJPL, Paredes SH, Law TF, de Lorenzo L, Feltcher ME, Finkel OM, Breakfield NW, Mieczkowski P, Jones CD, Paz-Ares J, Dangl JL. 2017. Root microbiota drive direct integration of phosphate stress and immunity. Nature 543:513–518. doi:10.1038/nature21417. 28297714 PMC5364063

[18] Shi S, Nuccio E, Herman DJ, Rijkers R, Estera K, Li J, Da Rocha UN, He Z, Pett-Ridge J, Brodie EL, Zhou J, Firestone M. 2015. Successional trajectories of rhizosphere bacterial communities over consecutive seasons. mBio 6:e00746-15. doi:10.1128/mBio.00746-15. 26242625 PMC4526712

[19] Edwards J, Johnson C, Santos-Medellín C, Lurie E, Podishetty NK, Bhatnagar S, Eisen JA, Sundaresan V. 2015. Structure, variation, and assembly of the root-associated microbiomes of rice. Proc Natl Acad Sci USA 112:E911–E920. doi:10.1073/pnas.1414592112. 25605935 PMC4345613

[20] Edwards JA, Santos-Medellín CM, Liechty ZS, Nguyen B, Lurie E, Eason S, Phillips G, Sundaresan V. 2018. Compositional shifts in root-associated bacterial and archaeal microbiota track the plant life cycle in field-grown rice. PLoS Biol 16:e2003862. doi:10.1371/journal.pbio.2003862. 29474469 PMC5841827

[21] Walters WA, Jin Z, Youngblut N, Wallace JG, Sutter J, Zhang W, González-Peña A, Peiffer J, Koren O, Shi Q, Knight R, Del Rio TG, Tringe SG, Buckler ES, Dangl JL, Ley RE. 2018. Large-scale replicated field study of maize rhizosphere identifies heritable microbes. Proc Natl Acad Sci USA 115:7368–7373. doi:10.1073/pnas.1800918115. 29941552 PMC6048482

[22] Peiffer JA, Spor A, Koren O, Jin Z, Tringe SG, Dangl JL, Buckler ES, Ley RE. 2013. Diversity and heritability of the maize rhizosphere microbiome under field conditions. Proc Natl Acad Sci USA 110:6548–6553. doi:10.1073/pnas.1302837110. 23576752 PMC3631645

[23] Wagner MR, Roberts JH, Balint‐Kurti P, Holland JB. 2020. Heterosis of leaf and rhizosphere microbiomes in field‐grown maize. New Phytol 228:1055–1069. doi:10.1111/nph.16730. 32521050

[24] Tai H, Lu X, Opitz N, Marcon C, Paschold A, Lithio A, Nettleton D, Hochholdinger F. 2016. Transcriptomic and anatomical complexity of primary, seminal, and crown roots highlight root type-specific functional diversity in maize (Zea mays L.). J Exp Bot 67:1123–1135. doi:10.1093/jxb/erv513. 26628518 PMC4753849

[25] Yu P, Wang C, Baldauf JA, Tai H, Gutjahr C, Hochholdinger F, Li C. 2018. Root type and soil phosphate determine the taxonomic landscape of colonizing fungi and the transcriptome of field-grown maize roots. New Phytol 217:1240–1253. doi:10.1111/nph.14893. 29154441

[26] Van Deynze A, Zamora P, Delaux P-M, Heitmann C, Jayaraman D, Rajasekar S, Graham D, Maeda J, Gibson D, Schwartz KD, Berry AM, Bhatnagar S, Jospin G, Darling A, Jeannotte R, Lopez J, Weimer BC, Eisen JA, Shapiro H-Y, Ané J-M, Bennett AB. 2018. Nitrogen fixation in a landrace of maize is supported by a mucilage-associated diazotrophic microbiota. PLoS Biol 16:e2006352. doi:10.1371/journal.pbio.2006352. 30086128 PMC6080747

[27] Rogers ED, Benfey PN. 2015. Regulation of plant root system architecture: implications for crop advancement. Curr Opin Biotechnol 32:93–98. doi:10.1016/j.copbio.2014.11.015. 25448235

[28] Gutjahr C, Casieri L, Paszkowski U. 2009. Glomus intraradices induces changes in root system architecture of rice independently of common symbiosis signaling. New Phytol 182:829–837. doi:10.1111/j.1469-8137.2009.02839.x. 19383099

[29] Brisson VL, Schmidt JE, Northen TR, Vogel JP, Gaudin ACM. 2019. Impacts of maize domestication and breeding on rhizosphere microbial community recruitment from a nutrient depleted agricultural soil. Sci Rep 9:15611. doi:10.1038/s41598-019-52148-y. 31666614 PMC6821752

[30] Jones DL, Nguyen C, Finlay RD. 2009. Carbon flow in the rhizosphere: carbon trading at the soil-root interface. Plant Soil 321:5–33. doi:10.1007/s11104-009-9925-0.

[31] Zhalnina K, Louie KB, Hao Z, Mansoori N, da Rocha UN, Shi S, Cho H, Karaoz U, Loqué D, Bowen BP, Firestone MK, Northen TR, Brodie EL. 2018. Dynamic root exudate chemistry and microbial substrate preferences drive patterns in rhizosphere microbial community assembly. Nat Microbiol 3:470–480. doi:10.1038/s41564-018-0129-3. 29556109

[32] Dombrowski N, Schlaeppi K, Agler MT, Hacquard S, Kemen E, Garrido-Oter R, Wunder J, Coupland G, Schulze-Lefert P. 2017. Root microbiota dynamics of perennial Arabis alpina are dependent on soil residence time but independent of flowering time. ISME J 11:43–55. doi:10.1038/ismej.2016.109. 27482927 PMC5097464

[33] Lundberg DS, Lebeis SL, Paredes SH, Yourstone S, Gehring J, Malfatti S, Tremblay J, Engelbrektson A, Kunin V, Del Rio TG, Edgar RC, Eickhorst T, Ley RE, Hugenholtz P, Tringe SG, Dangl JL. 2012. Defining the core Arabidopsis thaliana root microbiome. Nature 488:86–90. doi:10.1038/nature11237. 22859206 PMC4074413

[34] Wagner MR, Lundberg DS, Del Rio TG, Tringe SG, Dangl JL, Mitchell-Olds T. 2016. Host genotype and age shape the leaf and root microbiomes of a wild perennial plant. Nat Commun 7:12151. doi:10.1038/ncomms12151. 27402057 PMC4945892

[35] Bais HP, Weir TL, Perry LG, Gilroy S, Vivanco JM. 2006. The role of root exudates in rhizosphere interactions with plants and other organisms. Annu Rev Plant Biol 57:233–266. doi:10.1146/annurev.arplant.57.032905.105159. 16669762

[36] Walker TS, Bais HP, Grotewold E, Vivanco JM. 2003. Root exudation and rhizosphere biology. Plant Physiol 132:44–51. doi:10.1104/pp.102.019661. 12746510 PMC1540314

[37] Hu L, Robert CAM, Cadot S, Zhang X, Ye M, Li B, Manzo D, Chervet N, Steinger T, van der Heijden MGA, Schlaeppi K, Erb M. 2018. Root exudate metabolites drive plant-soil feedbacks on growth and defense by shaping the rhizosphere microbiota. Nat Commun 9:2738. doi:10.1038/s41467-018-05122-7. 30013066 PMC6048113

[38] Kudjordjie EN, Sapkota R, Steffensen SK, Fomsgaard IS, Nicolaisen M. 2019. Maize synthesized benzoxazinoids affect the host associated microbiome. Microbiome 7:59. doi:10.1186/s40168-019-0677-7. 30975184 PMC6460791

[39] Cotton TEA, Pétriacq P, Cameron DD, Al Meselmani M, Schwarzenbacher R, Rolfe SA, Ton J. 2019. Metabolic regulation of the maize rhizobiome by benzoxazinoids. ISME J 13:1647–1658. doi:10.1038/s41396-019-0375-2. 30796337 PMC6592824

[40] Willmann M, Gerlach N, Buer B, Polatajko A, Nagy R, Koebke E, Jansa J, Flisch R, Bucher M. 2013. Mycorrhizal phosphate uptake pathway in maize: vital for growth and cob development on nutrient poor agricultural and greenhouse soils. Front Plant Sci 4:533. doi:10.3389/fpls.2013.00533. 24409191 PMC3872827

[41] Fierer N. 2017. Embracing the unknown: disentangling the complexities of the soil microbiome. Nat Rev Microbiol 15:579–590. doi:10.1038/nrmicro.2017.87. 28824177

[42] Thiergart T, Durán P, Ellis T, Vannier N, Garrido-Oter R, Kemen E, Roux F, Alonso-Blanco C, Ågren J, Schulze-Lefert P, Hacquard S. 2020. Root microbiota assembly and adaptive differentiation among European Arabidopsis populations. Nat Ecol Evol 4:122–131. doi:10.1038/s41559-019-1063-3. 31900452

[43] Coleman-Derr D, Desgarennes D, Fonseca-Garcia C, Gross S, Clingenpeel S, Woyke T, North G, Visel A, Partida-Martinez LP, Tringe SG. 2016. Plant compartment and biogeography affect microbiome composition in cultivated and native Agave species. New Phytol 209:798–811. doi:10.1111/nph.13697. 26467257 PMC5057366

[44] Hartmann M, Frey B, Mayer J, Mäder P, Widmer F. 2015. Distinct soil microbial diversity under long-term organic and conventional farming. ISME J 9:1177–1194. doi:10.1038/ismej.2014.210. 25350160 PMC4409162

[45] Hartmann M, Widmer F. 2006. Community structure analyses are more sensitive to differences in soil bacterial communities than anonymous diversity indices. Appl Environ Microbiol 72:7804–7812. doi:10.1128/AEM.01464-06. 17041161 PMC1694274

[46] Robbins C, Thiergart T, Hacquard S, Garrido-Oter R, Gans W, Peiter E, Schulze-Lefert P, Spaepen S. 2018. Root-associated bacterial and fungal community profiles of Arabidopsis thaliana are robust across contrasting soil P levels. Phytobiomes J 2:24–34. doi:10.1094/PBIOMES-09-17-0042-R.

[47] Widmer F, Rasche F, Hartmann M, Fliessbach A. 2006. Community structures and substrate utilization of bacteria in soils from organic and conventional farming systems of the DOK long-term field experiment. Appl Soil Ecol 33:294–307. doi:10.1016/j.apsoil.2005.09.007.

[48] Birkhofer K, Bezemer TM, Bloem J, Bonkowski M, Christensen S, Dubois D, Ekelund F, Fließbach A, Gunst L, Hedlund K, Mäder P, Mikola J, Robin C, Setälä H, Tatin-Froux F, Van der Putten WH, Scheu S. 2008. Long-term organic farming fosters below and aboveground biota: implications for soil quality, biological control and productivity. Soil Biol Biochem 40:2297–2308. doi:10.1016/j.soilbio.2008.05.007.

[49] Fierer N, Bradford MA, Jackson RB. 2007. Toward an ecological classification of soil bacteria. Ecology 88:1354–1364. doi:10.1890/05-1839. 17601128

[50] Lapébie P, Lombard V, Drula E, Terrapon N, Henrissat B. 2019. Bacteroidetes use thousands of enzyme combinations to break down glycans. Nat Commun 10:2043. doi:10.1038/s41467-019-10068-5. 31053724 PMC6499787

[51] Fierer N, Jackson RB. 2006. The diversity and biogeography of soil bacterial communities. Proc Natl Acad Sci USA 103:626–631. doi:10.1073/pnas.0507535103. 16407148 PMC1334650

[52] Rousk J, Bååth E, Brookes PC, Lauber CL, Lozupone C, Caporaso JG, Knight R, Fierer N. 2010. Soil bacterial and fungal communities across a pH gradient in an arable soil. ISME J 4:1340–1351. doi:10.1038/ismej.2010.58. 20445636

[53] Richards TA, Soanes DM, Jones MDM, Vasieva O, Leonard G, Paszkiewicz K, Foster PG, Hall N, Talbot NJ. 2011. Horizontal gene transfer facilitated the evolution of plant parasitic mechanisms in the oomycetes. Proc Natl Acad Sci USA 108:15258–15263. doi:10.1073/pnas.1105100108. 21878562 PMC3174590

[54] Bai Y, Müller DB, Srinivas G, Garrido-Oter R, Potthoff E, Rott M, Dombrowski N, Münch PC, Spaepen S, Remus-Emsermann M, Hüttel B, McHardy AC, Vorholt JA, Schulze-Lefert P. 2015. Functional overlap of the Arabidopsis leaf and root microbiota. Nature 528:364–369. doi:10.1038/nature16192. 26633631

[55] Smith SE, Smith FA, Jakobsen I. 2003. Mycorrhizal fungi can dominate phosphate supply to plants irrespective of growth responses. Plant Physiol 133:16–20. doi:10.1104/pp.103.024380. 12970469 PMC1540331

[56] Luginbuehl LH, Menard GN, Kurup S, Van Erp H, Radhakrishnan GV, Breakspear A, Oldroyd GED, Eastmond PJ. 2017. Fatty acids in arbuscular mycorrhizal fungi are synthesized by the host plant. Science 356:1175–1178. doi:10.1126/science.aan0081. 28596311

[57] Keymer A, Pimprikar P, Wewer V, Huber C, Brands M, Bucerius SL, Delaux P-M, Klingl V, von Röpenack-Lahaye E, Wang TL, Eisenreich W, Dörmann P, Parniske M, Gutjahr C. 2017. Lipid transfer from plants to arbuscular mycorrhiza fungi. Elife 6:e29107. doi:10.7554/eLife.29107. 28726631 PMC5559270

[58] Bravo A, Brands M, Wewer V, Dörmann P, Harrison MJ. 2017. Arbuscular mycorrhiza-specific enzymes FatM and RAM2 fine-tune lipid biosynthesis to promote development of arbuscular mycorrhiza. New Phytol 214:1631–1645. doi:10.1111/nph.14533. 28380681

[59] Jiang Y, Wang W, Xie Q, Liu N, Liu L, Wang D, Zhang X, Yang C, Chen X, Tang D, Wang E. 2017. Plants transfer lipids to sustain colonization by mutualistic mycorrhizal and parasitic fungi. Science 356:1172–1175. doi:10.1126/science.aam9970. 28596307

[60] Fabiańska I, Pesch L, Koebke E, Gerlach N, Bucher M. 2020. Neighboring plants divergently modulate effects of loss-of-function in maize mycorrhizal phosphate uptake on host physiology and root fungal microbiota. PLoS One 15:e0232633. doi:10.1371/journal.pone.0232633. 32555651 PMC7299352

[61] Finkel OM, Salas-González I, Castrillo G, Spaepen S, Law TF, Teixeira PJPL, Jones CD, Dangl JL. 2019. The effects of soil phosphorus content on plant microbiota are driven by the plant phosphate starvation response. PLoS Biol 17:e3000534. doi:10.1371/journal.pbio.3000534. 31721759 PMC6876890

[62] IUSS Working Group WRB. 2015. World reference base for soil resources 2014, update 2015. International soil classification system for naming soils and creating legends for soil maps. World soil resources reports no 106. FAO, Rome, Italy.

[63] Unterseer S, Pophaly SD, Peis R, Westermeier P, Mayer M, Seidel MA, Haberer G, Mayer KFX, Ordas B, Pausch H, Tellier A, Bauer E, Schön C-C. 2016. A comprehensive study of the genomic differentiation between temperate Dent and Flint maize. Genome Biol 17:137. doi:10.1186/s13059-016-1009-x. 27387028 PMC4937532

[64] Abendroth LJ, Elmore RW, Boyer MJ, Markay SK. 2011. Corn growth and development. Iowa State University, Ames, IA.

[65] Bulgarelli D, Rott M, Schlaeppi K, Ver Loren van Themaat E, Ahmadinejad N, Assenza F, Rauf P, Huettel B, Reinhardt R, Schmelzer E, Peplies J, Gloeckner FO, Amann R, Eickhorst T, Schulze-Lefert P. 2012. Revealing structure and assembly cues for Arabidopsis root-inhabiting bacterial microbiota. Nature 488:91–95. doi:10.1038/nature11336. 22859207

[66] Callahan BJ, McMurdie PJ, Rosen MJ, Han AW, Johnson AJA, Holmes SP. 2016. DADA2: high-resolution sample inference from Illumina amplicon data. Nat Methods 13:581–583. doi:10.1038/nmeth.3869. 27214047 PMC4927377

[67] Wang Q, Garrity GM, Tiedje JM, Cole JR. 2007. Naïve Bayesian classifier for rapid assignment of rRNA sequences into the new bacterial taxonomy. Appl Environ Microbiol 73:5261–5267. doi:10.1128/AEM.00062-07. 17586664 PMC1950982

[68] Oksanen J, Blanchet FG, Friendly M, Kindt R, Legendre P, McGlinn D, Minchin PR, O’Hara RB, Simpson GL, Solymos P, Stevens MHH, Szoecs E, Wagner H. 2019. vegan: community ecology package.

