# Supplementary figures and images for: Maize Field Study Reveals Covaried Microbiota and Metabolic Changes in Roots over Plant Growth
Source: mBio. 2022 Mar 8;13(2):e02584-21. doi: 10.1128/mbio.02584-21 (PMC9040757; doi:10.1128/mbio.02584-21)

(a)

Bacteria

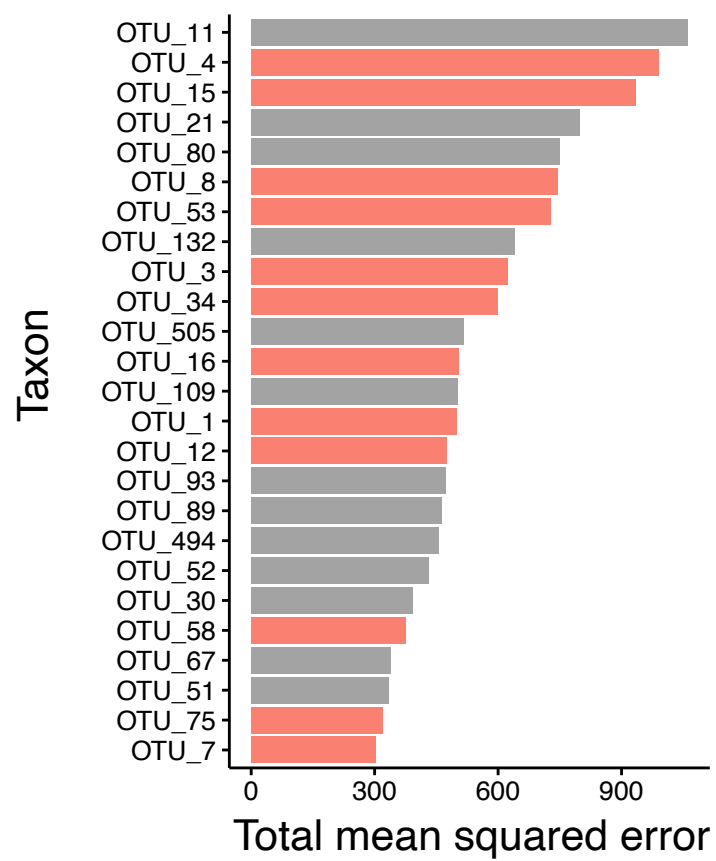

(b)

Fungi

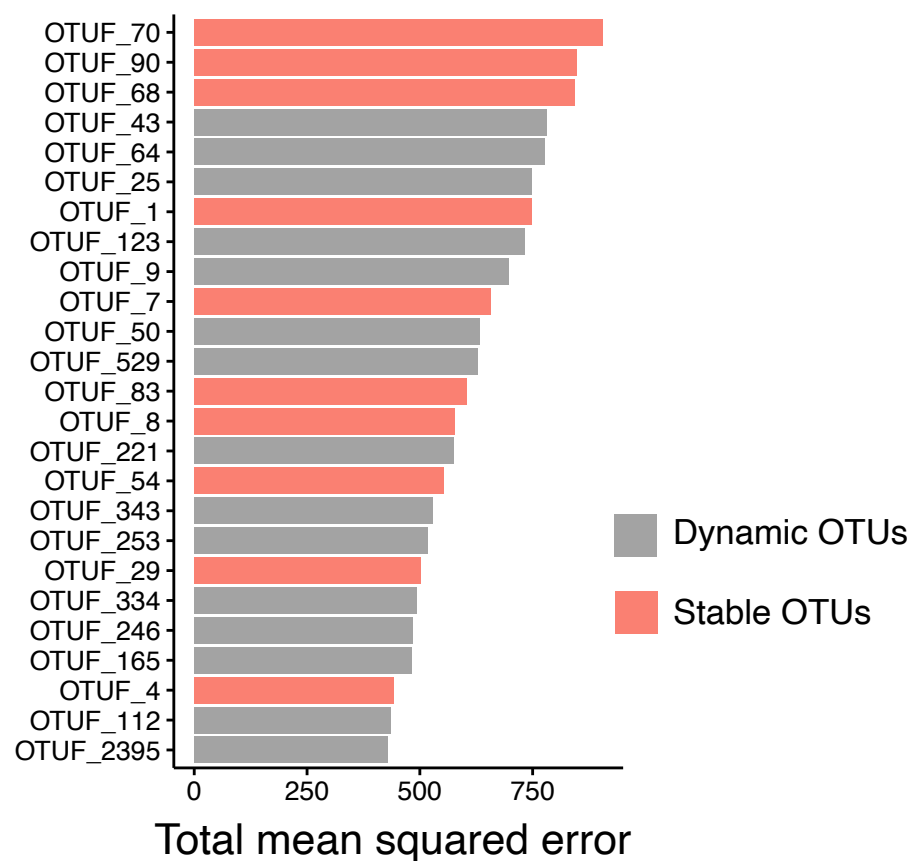

(c)

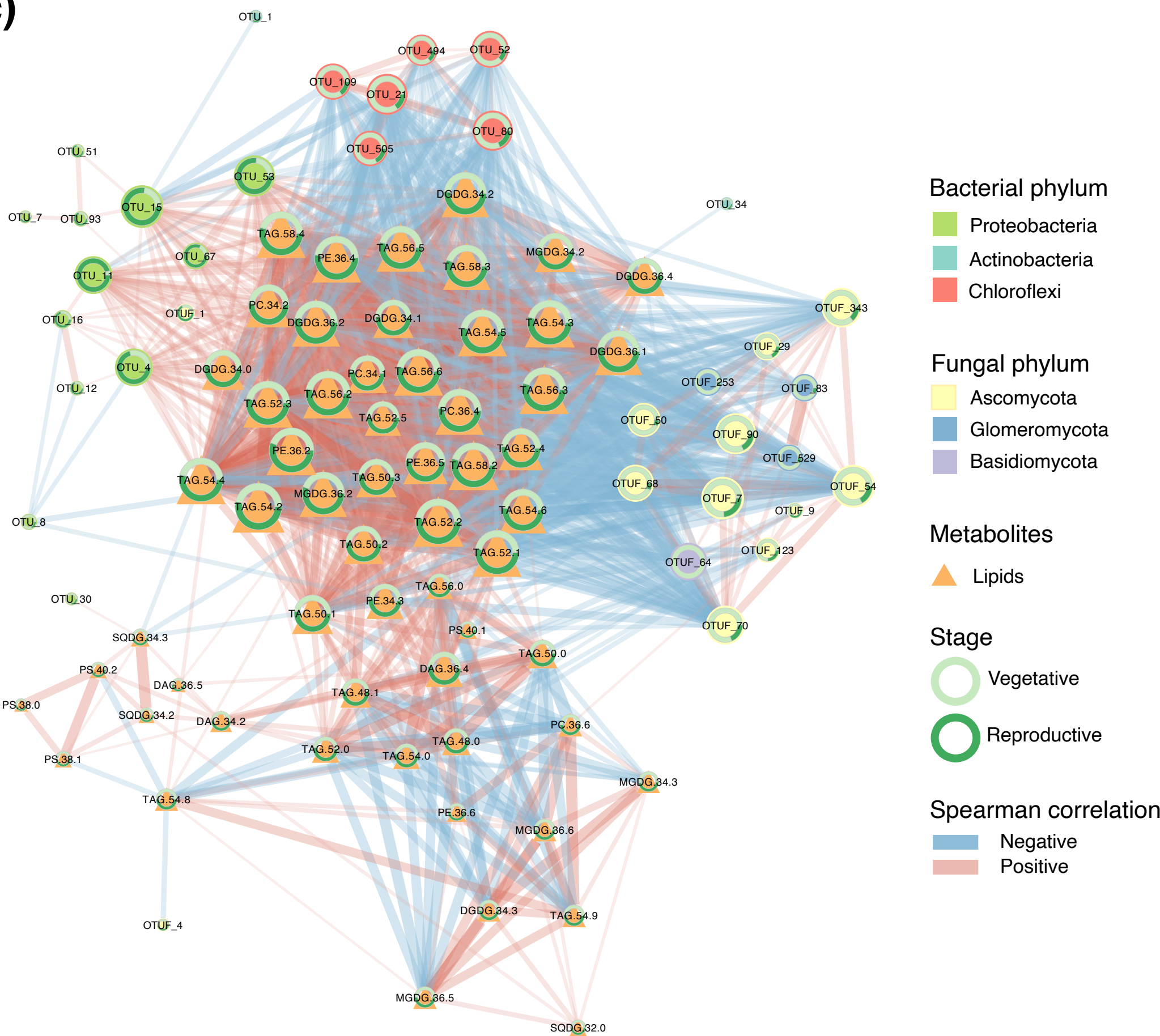

Supplement: FIG S7 [file mbio.02584-21-sf007.pdf]
