# Supplementary material for: Maize Field Study Reveals Covaried Microbiota and Metabolic Changes in Roots over Plant Growth
Source: mBio. 2022 Mar 8;13(2):e02584-21. doi: 10.1128/mbio.02584-21 (PMC9040757; doi:10.1128/mbio.02584-21)
Supplement: TABLE S1 [file mbio.02584-21-st001.pdf]

Table S1 Four different soil managements NK, NPK, CONMIN and BIODYN were applied on the DEMO (fertilization DEMOnstration experiment, Reckenholz) and DOK (Dynamic, organic and conventional managements, Therwil) long-term fields and corresponding soil parameters were measured before sowing. (a) N_{tot}: total nitrogen, N_{org}: organic nitrogen, N_{min}: mineralisable nitrogen. NK: N and K fertilization, NPK: N, P and K fertilization, CON: CONMIN (conventional mineral fertilization) and BIO: BIODYN (biodynamic). In the "Input" section the amount of complex compounds amended on soil is specified and the resulting amount of P and K is symbolized by the arrow. (b) Measurements were performed on the DEMO and DOK fields subjected to four different soil managements (NK, NPK, CONMIN, and BIODYN). pH H_2O: pH in aqueous solution, pH $CaCl_2$: pH in salt solution, C_{org}: organic carbon, N: total nitrogen, C/N: carbon/nitrogen ratio, POXC: labile organic carbon, sand content, silt content, clay content, LM: large macro-aggregates (> 2000 µm), SM: small macro-aggregates (250 - 2000 µm), µA: micro-aggregates (53 - 250 µm), S+C: silt and clay fraction (< 53 µm), CEC: cation exchange capacity, BS: base saturation, Mno: Mn(III)oxide content, Feo: short-range ordered Fe(III) oxide content, Fed: total FeIII oxide content, Mg: magnesium, K: potassium, Ca: calcium, P: phosphorus, Mn: manganese, Fe: iron, Cu: copper, Zn: zinc, Na: sodium, Mo: molybdenum, Al: aluminum, KW: aqua regia extractable elements). Mean and standard deviation (n = 6, for C_{org}, N, C/N, POXC; n = 12, for LM, SM, and µA; and n = 4 for S+C).

(a)

	DEMO NK	DEMO NPK	DOK CONMIN	DOK BIODYN
**Fertilization scheme**				
Farmyard manure (FYM)	-	-	-	30 m³/ha (autumn 2016)<br>Composted FYM + slurry (Jauche)
Mineral	N: ammonium sulphate (21% N, 24% S) / ammonium nitrate<br>K: potassium sulphate (50% K_2O, 18% S)<br>P: triple superphosphate (46% P_2O_5, 12% Ca)<br>Mg: Granumag (10% $MgSO_4$, 19% $MgCO_3$)	N: ammonium sulphate (21% N, 24% S) / ammonium nitrate (27% N)<br>K: potassium sulphate (50% K_2O, 18% S)<br>P: triple superphosphate (46% P_2O_5, 12% Ca)<br>Mg: Granumag (10% $MgSO_4$, 19% $MgCO_3$)	Synthetic (NPK) according to Swiss fertilization ordination	-
**Input**				
Dry matter	-	-	-	1020 kg/ha
Organic matter	-	-	-	720 kg/ha
N_{tot}	N_{min} in soil: 90 kg N/ha (02.06.17)<br>Ammonium sulphate: 55 kg N/ha (13.06.17)<br>Ammonium nitrate: 45 kg/ha (22.06.17)	N_{min} in soil: 90 kg N/ha (02.06.17)<br>Ammonium sulphate: 55 kg N/ha (13.06.17)<br>Ammonium nitrate: 45 kg/ha (22.06.17)	40 kg N/ha (15.06.17)<br>Calcium ammonium nitrate-20%	32 kg/ha
N_{org}	-	-	-	19 kg/ha
P	0 kg P_2O_5/ha --> 0 kg P/ha	75 kg P_2O_5/ha --> 33 kg P/ha	Not added in 2017	10 kg/ha
K	240 kg K_2O/ha --> 200 kg K/ha	240 kg K_2O/ha --> 200 kg K/ha	Not added in 2017	69 kg/ha
Ca / CaO	-	No special Ca and lime fertilizer<br>(12% Ca in triple superphosphate)	26 kg/ha<br>Calcium ammonium nitrate-13%	30 kg/ha
Mg	-	20 kg Mg/ha	9 kg/ha<br>Calcium ammonium nitrate-4.5%	11 kg/ha
S	Parts N and K fertilizer	Parts N and K fertilizer	-	-
**Plant protection scheme**				
Weed control	Mechanical<br>(no herbicides due to sensitivity of the inbred lines)	Mechanical<br>(no herbicides due to sensitivity of the inbred lines)	Mechanical and herbicides	Mechanical
Disease control	-	-	Chemical (damage threshold)	Indirect methods
Insect control	Trichogramma<br>(European corn borer)	Trichogramma<br>(European corn borer)	Chemical (damage threshold)	Plant extracts, biocontrol
Special treatments	-	-	Plant growth regulators	Biodynamic preparations
Irrigation	-	-	20L/m² (19.06.17)	20L/m² (19.06.17)

(b)

			DEMO (Reckenholz)		DOK (Therwil)					
			NK fertilization	NPK fertilization	Mineral conventional fertilization (CONMIN)			Biodynamic fertilization (BIODYN)		
			NK	NPK	CONMIN-1	CONMIN-2	CONMIN-3	BIODYN-1	BIODYN-2	BIODYN-3
pH	pH_H2O	-	5.97 ± 0.13	6.85 ± 0.13	6.36 ± 0.11	5.91± 0.16	6.81± 0.14	6.93± 0.27	7.08± 0.15	7.02± 0.20
	pH_CaCl2	-	5.15 ± 0.17	5.94 ± 0.14	5.53 ± 0.20	4.86±0.13	5.74± 0.16	5.95±0.30	6.04±0.15	6.02±0.18
Organic matter	Corg	(g/Kg)	11.49 ± 0.65	15.51 ± 0.35	14.66 ± 0.29	12.90±0.48	18.29± 0.50	17.26±0.85	17.65±0.49	22.36±0.52
	N	(g/Kg)	1.68 ± 0.10	2.16 ± 0.06	1.98 ± 0.06	1.73±0.09	2.40±0.06	2.18±0.07	2.25±0.09	2.83±0.08
	C/N	-	6.82 ± 0.21	7.19 ± 0.11	7.42 ± 0.26	7.46±0.23	7.64±0.15	7.91±0.17	7.85±0.18	7.91±0.18
	POXC	(mg/Kg)	207.46 ± 31.36	404.28 ± 28.52	451.83 ± 38.48	263.37±20.42	423.11±48.06	488.61±42.72	525.21±26.44	577.32±35.26
Particle size distribution	Sand	(g/Kg)	450.77 ± 10.43	390.45 ± 8.95	53.21 ± 1.66	54.67±1.98	30.18±4.79	55.37±1.47	53.23±2.55	24.83±1.09
	Silt	(g/Kg)	433.48 ± 8.25	485.38 ± 6.21	833.82 ± 5.56	840.14±2.10	822.77±3.36	840.01±1.19	836.73±3.29	830.24±1.28
	Clay	(g/Kg)	115.75 ± 2.34	124.17 ± 2.81	112.97 ± 4.78	105.19±3.20	147.05±2.92	104.63±1.51	110.04±4.17	144.93±0.99
Soil aggregation	LM	%	14.05 ± 4.31	16.83 ± 4.33	6.34 ± 4.40	2.31±1.82	8.62±5.07	4.63±1.76	2.95±1.05	10.97±4.76
	SM	%	42.22 ± 5.38	48.17 ± 2.47	50.03 ± 5.43	41.60±4.52	39.98±4.00	46.49±2.43	55.35±8.00	49.88±6.03
	µA	%	38.25 ± 3.85	30.25 ± 2.61	29.58 ± 8.89	38.10±3.98	37.37±5.51	30.08±1.47	26.74±6.56	27.48±7.79
	S+C	%	5.48 ± 1.18	4.74 ± 0.39	13.97 ± 1.12	17.93±3.71	14.02±1.74	18.80±0.56	14.96±2.37	11.60±3.02
Nutrient retention	CEC	($cmol_c$/Kg)	14.42 ± 2.21	16.58 ± 0.99	12.71 ± 0.30	9.69±0.31	19.69±0.33	13.67±0.82	14.91±0.29	20.62±0.23
	BS	%	76.21 ± 14.12	94.78 ± 4.54	105.16 ± 5.40	102.92±4.43	95.06±1.36	103.58±4.40	104.04±3.19	96.91±2.81
Oxides	Mno	(g/Kg)	0.39 ± 0.03	0.46 ± 0.02	0.44 ± 0.05	0.41±0.03	0.40±0.01	0.40±0.04	0.46±0.09	0.37±0.02
	Feo	(g/Kg)	2.18 ± 0.09	2.29 ± 0.06	2.51 ± 0.10	2.27±0.03	3.10±0.07	2.33±0.08	2.63±0.10	3.13±0.09
	Fed	(g/Kg)	8.44 ± 0.23	9.38 ± 0.21	8.11 ± 0.28	7.08±0.12	10.88±0.29	7.12±0.21	7.83±0.07	10.36±0.56
Plant available nutrients	Mg	(mg/Kg)	11.13 ± 4.05	16.83 ± 1.99	15.94 ± 2.15	14.58±0.70	16.78±2.09	19.24±4.48	20.35±2.69	22.71±2.88
	K	(mg/Kg)	10.77 ± 5.10	6.10 ± 1.01	15.77 ± 4.81	17.47±6.39	7.72±2.52	17.43±5.98	18.65±6.02	11.32±2.20
	Ca	(mg/Kg)	57.63 ± 19.13	142.56 ± 23.09	102.86 ± 9.64	70.90±5.98	149.71±24.88	157.55±34.14	163.57±15.79	181.18±19.27
	P	(mg/Kg)	0.95 ± 0.48	1.32 ± 0.21	2.20 ± 0.43	2.20±0.52	1.27±0.29	2.97±0.89	3.05±0.74	2.68±0.27
	Mn	(mg/Kg)	1.98 ± 0.67	1.41 ± 0.20	2.01 ± 0.33	3.18±0.49	1.67±0.23	1.58±0.26	1.25±0.15	1.10±0.15
	Fe	(mg/Kg)	17.65 ± 12.67	2.31 ± 1.05	8.32 ± 1.93	11.67±2.34	1.79±0.94	2.34±0.91	3.53±1.53	2.59±1.15
	Cu	(mg/Kg)	0.11 ± 0.04	0.13 ± 0.02	0.13 ± 0.01	0.14±0.01	0.09±0.01	0.11±0.01	0.11±0.01	0.12±0.01
	Zn	(mg/Kg)	0.08 ± 0.05	0.02 ± 0.01	0.06 ± 0.01	0.09±0.02	0.02±0.00	0.04±0.01	0.04±0.01	0.04±0.01
	Na	(mg/Kg)	5.90 ± 2.05	6.36 ± 0.97	7.77 ± 1.60	6.25±1.48	7.91±1.87	8.18±1.25	8.49±2.01	11.88±2.85
	Mo	(mg/Kg)	0.00 ± 0.00	0.00 ± 0.00	0.00 ± 0.00	0.00±0.00	0.00 ±0.00	0.00±0.00	0.00±0.00	0.00±0.00
aqua-regia extractable elements	Mg_KW	(mg/Kg)	4263.90 ± 59.22	5191.82 ± 111.83	3593.33 ± 140.11	3322.90±83.40	5089.09±178.40	3189.94±70.09	3607.76±84.46	5011.35±172.10
	K_KW	(mg/Kg)	4873.71 ± 176.11	5883.75 ± 219.21	4449.99 ± 274.39	4175.58±303.73	6349.11±193.15	4072.33±82.48	4577.32±148.38	6168.40±222.35
	Ca_KW	(mg/Kg)	2090.19 ± 160.41	2958.86 ± 148.25	3192.51 ± 120.60	2625.98±162.90	3608.36±139.05	3049.04±142.90	3356.22±64.81	3718.40±156.35
	P_KW	(mg/Kg)	341.50 ± 17.93	487.43 ± 18.57	451.37 ± 29.85	441.48±21.92	536.92±21.95	422.11±21.24	482.49±14.36	571.17±15.91
	Mn_KW	(mg/Kg)	653.38 ± 23.73	720.00 ± 34.93	748.49 ± 70.75	653.20±43.78	673.54±19.72	717.00±58.12	680.30±66.39	623.08±25.63
	Fe_KW	(mg/Kg)	18750.89 ± 224.77	21163.55 ± 408.08	18515.74 ±677.35	17016.41±436.30	24757.01±890.56	16778.96±442.74	18430.64±449.81	24335.84±901.78
	Cu_KW	(mg/Kg)	21.50 ± 0.64	33.02 ± 0.80	19.23 ± 0.96	19.57±1.47	24.93±0.80	17.18±0.53	18.16±0.56	25.07±1.11
	Zn_KW	(mg/Kg)	46.49 ± 2.18	53.34 ± 1.44	50.70 ± 1.55	45.30±1.39	65.33±2.10	49.18±1.37	53.38±1.47	69.97±2.38
	Al_KW	(mg/Kg)	23817.27 ± 491.56	26869.82 ± 741.26	20926.41 ± 706.91	19208.92±713.04	30127.89±1047.27	18802.24±494.79	20757.97±402.32	29226.33±1044.54
	Mo_KW	(mg/Kg)	0.74 ± 0.03	0.78 ± 0.04	0.40 ± 0.02	0.36±0.04	0.43±0.02	0.41±0.02	0.46±0.02	0.52±0.04
