# Supplementary material for: Maize Field Study Reveals Covaried Microbiota and Metabolic Changes in Roots over Plant Growth
Source: mBio. 2022 Mar 8;13(2):e02584-21. doi: 10.1128/mbio.02584-21 (PMC9040757; doi:10.1128/mbio.02584-21)
Supplement: TEXT S1 [file mbio.02584-21-t0001.docx]

**Methods_S1**

1. **Field experiment and sampling**

The experiment was performed on two long-term fields: DEMO (47°25′31″ N, 8°30′59″ E; MAT 9.4 °C, MAP 1031 mm; and DOK (47°30′09″ N, 7°32′21″ E; MAT 10.5 °C, MAP 842 mm), located in Switzerland. The DOK field includes eight different soil management conditions (Hartmann *et al.*, 2015), among which only CONMIN and BIODYN were used in this study. All the plant samples were harvested between 13:00 and 15:00 hrs to ensure the stability of plant metabolites. In total, we collected 1,104 samples, including 60 bulk soil samples before sowing (6 per plot), 528 samples at vegetative and 528 samples at reproductive stage. At each plant growth stage, three plants per genotype and per plot were harvested (120 plants at each growth stage) and three soil samples were collected per plot. For each plant, root samples were fractionated in root and rhizosphere. Each sample was amplified for bacterial, fungal and oomycetal community profiling.

1. **Analysis of soil properties**

The analyses of bulk soil before sowing were performed after an air-drying step in the laboratory, on soil that was partly sieved (<2 mm) and partly grinded in a mixer mill. The soil pH was measured potentiometrically by a glass electrode in a 0.01 mol L^–1^ calcium chloride (CaCl_2_) solution mixed 5:1 with soil (v/v). The particle size distribution (sand, silt, clay content) was determined using a laser particle analyzer (LS13 320, Beckman Coulter). The organic carbon (Corg) and total nitrogen (N) were measured by dry combustion with a CNS analyzer (Vario EL cube, Elementar). The permanganate-oxidizable carbon (POXC) was obtained according to the method of Weil *et al*. (Weil *et al.*, 2003). The effective cation exchange capacity (CEC) was determined using a hexamine cobalt (III) chloride solution according to ISO 23470:2007. The plant-available nutrients (Mg, K, Ca, P, Mn, Fe, Cu, Zn, Na, Mo) were extracted with CO_2_-saturated water mixed 2.5:1 with soil (v/v) and pseudo-total element (Mg, K, Ca, P, Mn, Fe, Cu, Zn, Al, Mo) concentrations were determined after extraction with aqua-regia, and were measured by inductively coupled plasma mass spectrometry (XSeries 2, Thermo Scientific).

**Root lipid profiling**

Lipids were extracted as described before (Hummel *et al.*, 2011). Briefly, 100 mg of frozen roots tissue homogenized into a thin powder and lipids were extracted with 1 mL of precooled (−20°C) extraction buffer (methanol/methyl-tert-butyl-ether [1:3, v/v] mixture, spiked with 0.1 μg/mL of PE 34:0 [17:0, 17:0] as internal standard. After 10 min incubation in 4°C and sonication for 10 min in a sonic bath, 500 μL of water/methanol mixture was added. Samples were centrifuged (5 min, 14 000 g) to separate the lipophilic and polar phases. Aliquots of 500 μL from the lipophilic phase were collected and dried under vacuum. The dried lipids were re-suspended in 300 μL UPLC-grade acetonitrile:isopropanol (7:3 v/v) mixture, followed by vortexing and transferring to a glass vial. The samples were then processed using ultra-performance liquid chromatography coupled with Fourier transform mass spectrometry (UPLC-FT-MS). Two microliters were injected on a C8 reversed phase column (100 mm × 2.1 mm × 1.7 μm particle size, Waters) at 60°C, using a Waters Acquity UPLC system. The two mobile phases consisted of Buffer A: 1% 1 M NH4Ac, 0.1% acetic acid in water (UPLC MS grade, BioSolve), and Buffer B: acetonitrile:isopropanol (7:3, UPLC grade BioSolve) containing 1% 1 M NH4Ac, 0.1% acetic acid. The following gradient profile was applied: 1 min 45% A, 3 min linear gradient from 45% A to 35% A, 8 min linear gradient from 25% to 11% A, 3 min linear gradient from 11% to 1% A. Finally, after washing the column for 3 min with 1% A the buffer was set back to 45% A and the column was re-equilibrated for 4 min (total run time of 22 min). The flow rate of the mobile phase was 400 μL/min.

The mass spectra were acquired using an Exactive mass spectrometer (Thermo-Fisher, Bremen, Germany) equipped with an ESI interface. The spectra were recorded using altering full scan and all-ion fragmentation scan mode, covering a mass range from 100–1500 m/z. The resolution was set to 10,000 with 10 scans per second, restricting the Orbitrap loading time to a maximum of 100 ms with a target value of 1E6 ions. The capillary voltage was set to 3 kV with a sheath gas flow value of 60 and an auxiliary gas flow of 35. The capillary temperature was set to 150°C, while the drying gas in the heated electro spray source was set to 350°C. The skimmer voltage was held at 25 V while the tube lens was set to a value of 130 V. The spectra were recorded from min 1 to min 20 of the UPLC gradients (Hummel *et al.*, 2011). Lipids were analyzed exactly as described in Garbowicz *et al.*, 2018. Shortly, processing of chromatograms, peak detection and integration were performed using REFINER MS 10.0 (GeneData, http://www.genedata.com). Processing of mass spectrometry data included the removal of the fragmentation information, isotopic peaks, as well as chemical noise. Obtained features (m/z at a certain retention time) were queried against an in-house lipid database for further annotation. MS/MS fragmentation using collision-induced dissociation mass spectra (25 eV collision energy) was used for further validation of representatives of different lipid classes. The database used in this project includes nearly 200 lipid species of the following classes: diacylglycerols (DAGs), digalactosyldiacylglycerols (DGDGs), monogalactosyldiacylglycerols (MGDGs), phosphatidylcholines (PCs), phosphatidylethanolamines (PEs), phosphatidylglycerols (PGs), phosphatidylserines (PSs), phosphatidylinositols (PIs), sulfoquinovosyl diacylglycerols (SQDGs), and triacylglycerides (TAGs). The lipids reporting list containing, among others, information of retention time and m/z follows the recommendations described by Fernie *et al.*, 2011 and is presented in Supplementary Dataset.

1. **Determination of soluble sugars, starch and free amino acids from root samples**

For extraction of amino acids and sugars ca. 50mg of roots fresh weight were heated for 60 min at -80ºC with 500 µL ethanol. After cooling for 15 min and centrifugation for 5 min at 10000 × g supernatants were dried for 60-90min in a vaccum concentrator. The dried aliquots were subsequently re-suspended in 250µl water.

For amino acids quantification 10 µL of the extracts were derivatized using the fluoropohore 6-aminoquinolyl-N-hydroxysuccimidyl carbamate (AQC) (Cohen & Michaud, 1993) and 10 µL of this solution was subsequently analyzed using a Summit reversed phase HPLC-system (Dionex) with a fluorescence detector RF2000 (excitation/emission λ=300/400nm, Dionex) as described by Hofmann, 2011. In brief, for separation a Luna C18(2) column (5µm, 250x 4,6mm) with a security guard pre-column (4.0x 3.0mm) (Phenomenex) was used. Amino acids were eluted with a trimeric gradient of buffer A (140 mM sodium acetate, 7 mM triethanolamin, pH6,2), eluent B (Acetonitril), and eluent C (water) within a total run time of 60 min at a flow rate of 1 mL min^-1^ and a column temperature of 37ºC as follows: 0 to 0.5 min, 4% B; 0.5 to 43.5 min, 20.5% B; 43.5 to 50 min, 27% B; 50 to 51.5 min, 35% B; 51.5 to 52.5 min, 60% B/40% C; 52.5 to 55.5 min, 60% B/40% C; 55.5 to 59 min, 100% A; 59 to 60 min, 100% A. The contents of amino acids were calculated based on peak areas relative to pure standards in a range of 4-200 nmol. Retention times in minutes were as follows: Asp 9.6, Glu 11.1, Asn 15.9, Ser 16.4, Gln 17.4, Gly 17.9, His 18.3, Thr 20.1, Arg 21.4, Ala 22.5, Pro 25.0, Tyr 32.2, Val 34.9, Ile 42.2, Lys 42.7, Leu 43.5, Phe 46,4.

Soluble sugars and starch were determined enzymatically using the ethanolic extracts described above and a microtiter plate reader (BioTek, Bad Friedrichshall, Germany) following Kogel *et al.* (2010). In brief, glucose, fructose, and sucrose were determined together in a coupled optical test through the sequential addition of coupling enzymes (hexokinase, phosphoglucoisomerase, and invertase) and the stoichometric conversion of NAD^+^ to NADH at λ = 340 nm. Concentrations were calculated from the difference of the end- and start-values of optical density, together with the extinction coefficient for NADH applying the Lambert-Beer law. For the insoluble starch fraction, the glucose content was measured after the conversion with amylogycosidase.

1. **Total elemental composition of roots**

Maize roots were homogenized and approximately 100 mg of dry matter were used for acid digestion in 3 ml of 67% nitric acid (HNO_3_) for 2 hours at 100°C. Afterwards, the solutions were diluted with demineralised water and filtered (Whatman® filter grade 1, GE Healthcare Life Sciences, USA) to exclude solid particles. Elemental composition was determined with an Agilent 7700 ICP‐MS (Agilent, http://www.home.agilent.com), following the manufacturer's instructions.

1. **Root mycorrhizal colonization**

Fine roots were first cut into 1-cm-long pieces and then heated for 15 min in 10% KOH at 95°C. After two rinses with water, roots were transferred to ink solution (5% acetic acid, 5% ink) and incubated for 3 min at 95°C. After rinsing in water for destaining, roots were transferred to 20% acetic acid solution for 10 min at 75°C. The destaining step was performed three times in total. Then, roots were washed and stored in water. The microscopic analysis was based on a magnified intersection method (McGonigle *et al.*, 1990). Briefly, ten root fragments of about 1 cm in length were placed onto microscopic slides. At 20x magnification, 100 views were observed and classified into categories: (I) no colonization visible, (II) hyphae, (III) hyphae and arbuscule, and (IV) hyphae and arbuscule and vesicle. Finally, the degree of mycorrhizal colonization was expressed as a percentage by adding together the categories including arbuscules (hyphae + arbuscules, hyphae + arbuscules + vesicles) and dividing by the total number of views.

1. **Root and rhizosphere fractionation**

The rhizosphere fraction was obtained after a first washing step of around 1g of root material in sterile falcon tubes with 5 ml PBS-Silwet L-77 (PBS-S) using a cycle agitator (20 min, 35 rpm) and centrifugation (20 min, 4000x*g*, 16°C). The root fraction was obtained after two more wash steps in PBS-S and in TE 1x buffer supplemented with 0.1% Triton X-100 (TE-T), respectively, and sonication (10 cycles: 30 sec pulse 160 W, 30 sec break). The roots were washed in TE-T, dried briefly on Whatman paper, frozen, and crushed using 10 sterile metal beads of 3.2 mm (-26°C, 6500 rpm, 2x 30 sec, 15 sec break) (Precellys 24-Cryolys tissue lyzer (Bertin Technologies, Montigny-le- Bretonneux, France).

1. **Total genomic DNA extraction and amplicon sequencing**

During DNA extraction a first lysis step was performed at 6300 rpm for 45 sec (Precellys 24 tissue lyzer (Bertin Technologies, Montigny-le- Bretonneux, France). Samples were first amplified in triplicate, digested, and then amplified in a second PCR by primers containing barcodes and Illumina adaptors. After pooling of triplicate reactions, the quality of amplification was checked on agarose gels (5 µL, 1.5 %, 100 V, 30 minutes). Fungal and oomycetal PCR products were directly purified twice by Ampure XP beads (Agencourt, Beckman Coulter, USA). All bacterial PCR products were analyzed on agarose gels (100 µL, 2H, 80V, 1.5%) to separate bacterial products from plant mitochondrial products. Bands corresponding to 500 bp were cut and then purified (QIAquick gel extraction kit, QIAGEN, Hilden, Germany). After checking DNA concentration (Quant-IT^TM^ Picogreen, Invitrogen, Oregon, USA), PCR products belonging to the same microbial kingdoms were pooled together in equimolar amounts to build one library per kingdom. Each library was purified twice by Ampure XP beads (Agencourt, Beckman Coulter, USA), quantified by Qubit, and finally pooled together in equimolar amounts (or a 5:1 for bacteria:fungi ratio if needed).

1. **Amplicon data processing**

The sequenced amplicon profiling data were processed with workflow based on DADA2 (v1.12.1, <https://github.com/Guan06/DADA2_pipeline>) (Callahan *et al.*, 2016). Forward and reverse reads were demultiplexed. For bacterial samples, raw sequencing reads were subsequently truncated to 260 bp (forward) or 240 bp (reverse) and filtered with “maxN=0, maxEE=c(2,2), truncQ=2, rm.phix=TRUE”. For fungal and oomycetal communities, we mapped the primers to the sequencing reads to trim the non-amplified region and then filtered with “maxN=0, maxEE=c(2, 2)”. After learning the error rates, ASVs were generated by merging the corrected forward and reverse reads, and chimeras were removed.

1. **Community diversity analysis at the phylum level**

Relative abundance of ASVs belonging to the same phylum were summed up to obtain the relative abundance of the corresponding phylum, and based on this, the BC matrix was calculated. Mean BC between each sample, and samples from unplanted soil (the most soil-like condition, *x_n_*) and reproductive-stage root (the most root-like condition, *y_n_*) were calculated. Samples with minimum *x_n_* were defined as the starting point (*x_0_*, *y_0_*) of the community and the Euclidean distance between this sample and all other samples was calculated. Additionally, the relative abundance of each phylum was compared between conditions by analysis of variance (ANOVA) and Tukey’s post-hoc test (De Mendiburu & Yassen, 2020).

1. **Biomass, metabolites, and total elements data processing**

Euclidean distances between samples were calculated based on biomass, metabolic and ionomic profiles (data could be found on GitHub in folder <https://github.com/Guan06/Bourceret_and_Guan_et_al_2021/tree/main/00.data/meta_data>). The explained variance was computed with model “~ Management * Stage” for four inbred lines, respectively, and “~ Management * Stage * Genotype” for the comparison between wild type and mutant lines.

1. **Predictive taxa for root lipid identification and network construction**

To identify the predictive taxa for root lipid dynamics, we used function ‘ramdomForest()’ from R package randomForest (Liaw & Wiener, 2002) with parameter ‘ntree = 1000’ using OTU table (for bacteria and fungi respectively) and lipid profiling dataset in the model. Afterwards, mean squared error (MSE) was calculated with function ‘impartance()’ using ‘scale = TRUE’. Taxa with highest MSE (n = 25) was then extracted as predictive OTUs. We calculated Spearman correlation between predictive OTUs and lipids and filtered the correlation matrix by keeping only significant (*P* < 0.05) and strong (with absolute value > 0.5) coefficients. Subsequently, the network was visualized by Cytoscape (v 3.8.2, Shannon, 2003).

**References**

**Cohen SA, Michaud DP**. **1993**. Synthesis of a Fluorescent Derivatizing Reagent, 6-Aminoquinolyl-N-Hydroxysuccinimidyl Carbamate, and Its Application for the Analysis of Hydrolysate Amino Acids via High-Performance Liquid Chromatography. *Analytical Biochemistry* **211**: 279–287.

**Fernie AR, Aharoni A, Willmitzer L, Stitt M, Tohge T, Kopka J, Carroll AJ, Saito K, Fraser PD, DeLuca V**. **2011**. Recommendations for Reporting Metabolite Data. *The Plant Cell* **23**: 2477–2482.

**Garbowicz K, Liu Z, Alseekh S, Tieman D, Taylor M, Kuhalskaya A, Ofner I, Zamir D, Klee HJ, Fernie AR, *et al.*** **2018**. Quantitative Trait Loci Analysis Identifies a Prominent Gene Involved in the Production of Fatty Acid-Derived Flavor Volatiles in Tomato. *Molecular Plant* **11**: 1147–1165.

**Hartmann M, Frey B, Mayer J, Mäder P, Widmer F**. **2015**. Distinct soil microbial diversity under long-term organic and conventional farming. *The ISME Journal* **9**: 1177–1194.

**Hedley MJ, Stewart JWB, Chauhan BS**. **1982**. Changes in Inorganic and Organic Soil Phosphorus Fractions Induced by Cultivation Practices and by Laboratory Incubations. *Soil Science Society of America Journal* **46**: 970–976.

**Hofmann J**. **2011**. Detecting functional groups of Arabidopsis mutants by metabolic profiling and evaluation of pleiotropic responses. *Frontiers in Plant Science* **2**: 1–13.

**Hummel J, Segu S, Li Y, Irgang S, Jueppner J, Giavalisco P**. **2011**. Ultra Performance Liquid Chromatography and High Resolution Mass Spectrometry for the Analysis of Plant Lipids. *Frontiers in Plant Science* **2**.

**Kogel K-H, Voll LM, Schafer P, Jansen C, Wu Y, Langen G, Imani J, Hofmann J, Schmiedl A, Sonnewald S, *et al.*** **2010**. Transcriptome and metabolome profiling of field-grown transgenic barley lack induced differences but show cultivar-specific variances. *Proceedings of the National Academy of Sciences* **107**: 6198–6203.

**Liaw A, Wiener M**. **2002**. Classification and Regression by randomForest. *R News* **2**: 18–22.

**McGonigle TP, Miller MH, Evans DG, Fairchild GL, Swan JA**. **1990**. A new method which gives an objective measure of colonization of roots by vesicular-arbuscular mycorrhizal fungi. *New Phytologist* **115**: 495–501.

**Shannon P**. **2003**. Cytoscape: A Software Environment for Integrated Models of Biomolecular Interaction Networks. *Genome Research* **13**: 2498–2504.

**Weil RR, Islam KR, Stine MA, Gruver JB, Samson-Liebig SE**. **2003**. Estimating active carbon for soil quality assessment: A simplified method for laboratory and field use. *American Journal of Alternative Agriculture* **18**: 3–17.
